# Macrofaunal Patterns in and around du Couedic and Bonney Submarine Canyons, South Australia

**DOI:** 10.1371/journal.pone.0143921

**Published:** 2015-11-30

**Authors:** Kathleen E. Conlan, David R. Currie, Sabine Dittmann, Shirley J. Sorokin, Ed Hendrycks

**Affiliations:** 1 Canadian Museum of Nature, Ottawa, Ontario, Canada; 2 South Australian Research and Development Institute (Aquatic Sciences), Adelaide, South Australia, Australia; 3 Flinders University, Adelaide, South Australia, Australia; Instituto Español de Oceanografía, SPAIN

## Abstract

Two South Australian canyons, one shelf-incising (du Couedic) and one slope-limited (Bonney) were compared for macrofaunal patterns on the shelf and slope that spanned three water masses. It was hypothesized that community structure would (H1) significantly differ by water mass, (H2) show significant regional differences and (H3) differ significantly between interior and exterior of each canyon. Five hundred and thirty-one species of macrofauna ≥1 mm were captured at 27 stations situated in depth stratified transects inside and outside the canyons from 100 to1500 m depth. The macrofauna showed a positive relationship to depth in abundance, biomass, species richness and community composition while taxonomic distinctness and evenness remained high at all depths. Biotic variation on the shelf was best defined by variation in bottom water primary production while sediment characteristics and bottom water oxygen, temperature and nutrients defined biotic variation at greater depth. Community structure differed significantly (p<0.01) among the three water masses (shelf-flowing South Australian current, upper slope Flinders current and lower slope Antarctic Intermediate Water) (H1). Although community differences between the du Couedic and Bonney regions were marginally above significance at p = 0.05 (H2), over half of the species captured were unique to each region. This supports the evidence from fish and megafaunal distributions that the du Couedic and Bonney areas are in different bioregions. Overall, the canyon interiors were not significantly different in community composition from the exterior (H3). However, both canyons had higher abundance and/or biomass, increased species dominance, different species composition and coarser sediments near the canyon heads compared to outside the canyons at the same depth (500 m), suggestive of heightened currents within the canyons that influence community composition there. At 1000–1500 m, the canyon interiors were depauperate, typical of V-shaped canyons elsewhere. The large number of species captured, given the relatively low sampling effort and focus on the larger macrofauna, support previous studies that identify the South Australian coast as a high biodiversity area.

## Introduction

Submarine canyons incise the slope and shelf of continents in all oceans and nearly 6000 are known to date [1]. They are focal points for upwelling, entraining benthopelagic prey, macrophyte detritus, particulates and nutrient-rich water to support a highly productive, species-rich and distinctive community [2–8]. Submarine canyons can bring deep water and neutrally buoyant matter to the surface with cyclonic water movement [9; 10]. They can also have their own internal eddies and funnel shelf water and sediments down-slope, modifying mixing patterns [1; 11; 12].

The Australian coast has 713 submarine canyons identified on the slope, of which 87% descend from the shelf edge or deeper and 13% incise the shelf as well [1; 4; 12–17]. The largest number (n = 187) and areal coverage (25,995 km^2^) of canyons occur on the southeast Australian coast [17]. These canyons can transport upwelled water onto the shelf from 500 m depth if upwelling is caused by winds, or much deeper (2000 m) as a result of cross-slope currents [10; 18]. These canyons may also be conduits for the transport of deep-water oil seeps to the surface from as deep as 4000 m [10].

Given that submarine canyons can enhance and otherwise modify the biological conditions within their interiors e.g., [1; 3–7; 19–22] and on the adjacent shelf [10], the southeast Australian coast may show extensive canyon effects. The du Couedic Canyon and associated 4310 km^2^ Murray canyon system (135°E—138.5°E; [15; 17]) is thought to support a diverse soft sediment ecosystem that may be enhanced by upwelling of nutrient-rich slope water [10; 23; 24]. Southern bluefin tuna (*Thunnus maccoyii*) congregate here during the austral summer-autumn upwelling season, feeding on sardine (*Sardinops sagax*) and anchovy (*Engraulis australis*) which spawn here at densities that are significantly higher than elsewhere in southern Australia [25].

The first biological assessment of du Couedic Canyon was by [26] for megafauna and compared with Bonney Canyon, 300 km to the east. The two canyons differed significantly, attributable to differences in location, oceanography and topography [26] and differing benthic bioregions [27; 28]. Enrichment in sponge biomass along the upper du Couedic Canyon axis was thought to be due to the canyon’s unique exposure to nutrient-rich outflows from neighbouring Spencer Gulf. Fish assessment in Bonney Canyon yielded no canyon-associated patterns but a strong depth gradient associated with three water masses, surface water (<450 m depth), the core of the Flinders Current (500 m) and Antarctic Intermediate Water (1000 m) [29]. The purpose of this paper is to compare the macrofauna between the two canyons and between their interiors and exteriors on the shelf and slope to 1500 m depth. Different patterns may occur with macrofauna because they are not widely motile compared to fish, are smaller and have higher turnover than megafauna and are integrators of environmental effects. Macrofauna are also usually more abundant and diverse *per* unit catch size so patterns can be based on a larger faunal number than for fish or megafauna. We hypothesize that the macrofauna (H1) will be zoned by depth and water mass but differ (H2) between the du Couedic and Bonney regions and (H3) between the interior and exterior of each canyon. The effects of canyons on macrofauna have been increasingly studied world-wide e.g. [19; 22; 30; 31]. Although some Australian canyons have been sampled for macrofauna as part of other surveys [32; 33], this study is the first to systematically sample and report macrofaunal patterns in Australian canyons.

## Materials and Methods

### Study area

**Fig 1:**
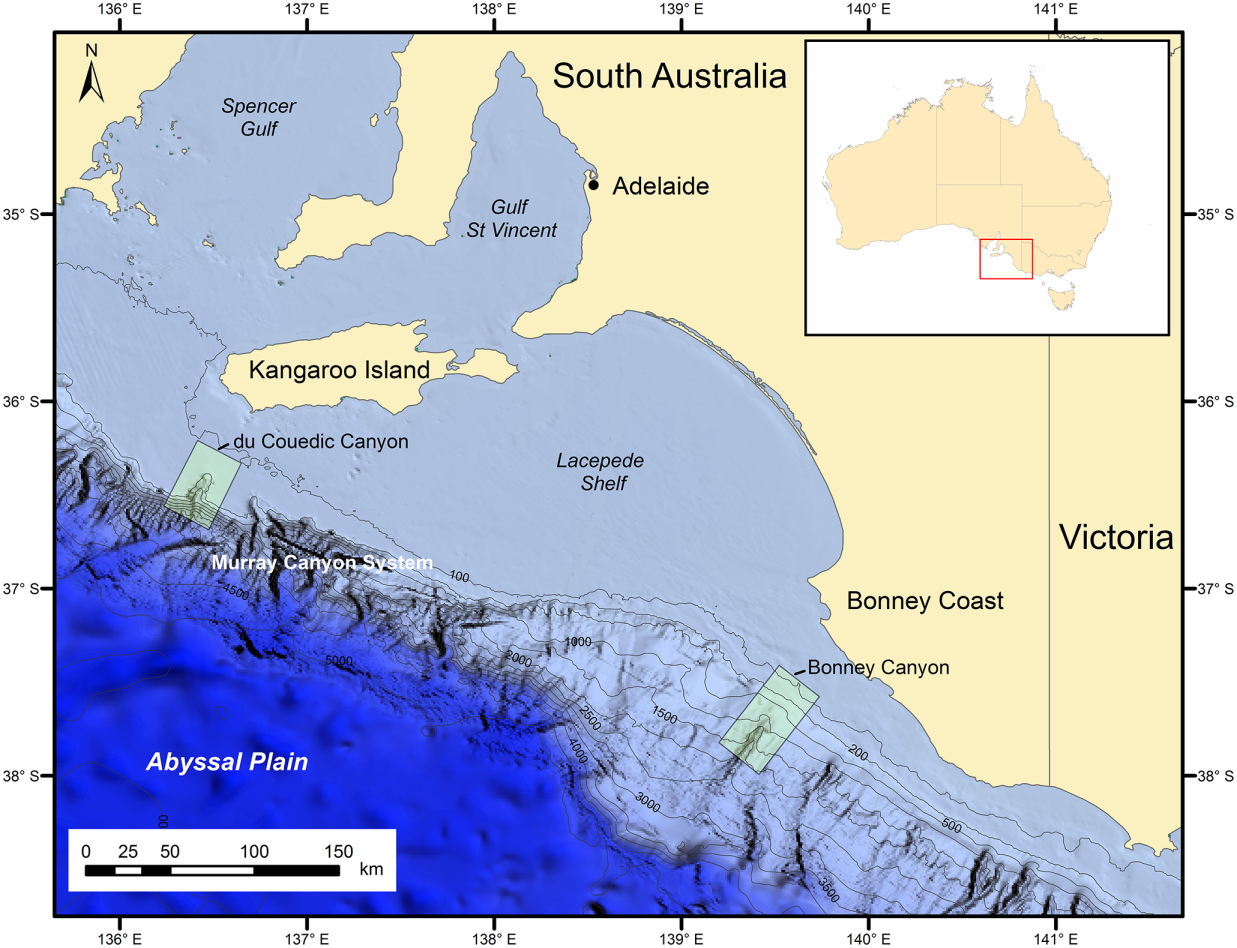
Bathymetry of the South Australian coast showing the two regions sampled (rectangles), containing the shelf-incising du Couedic Canyon to the west and the slope-confined Bonney Canyon to the east.

**Fig 2:**
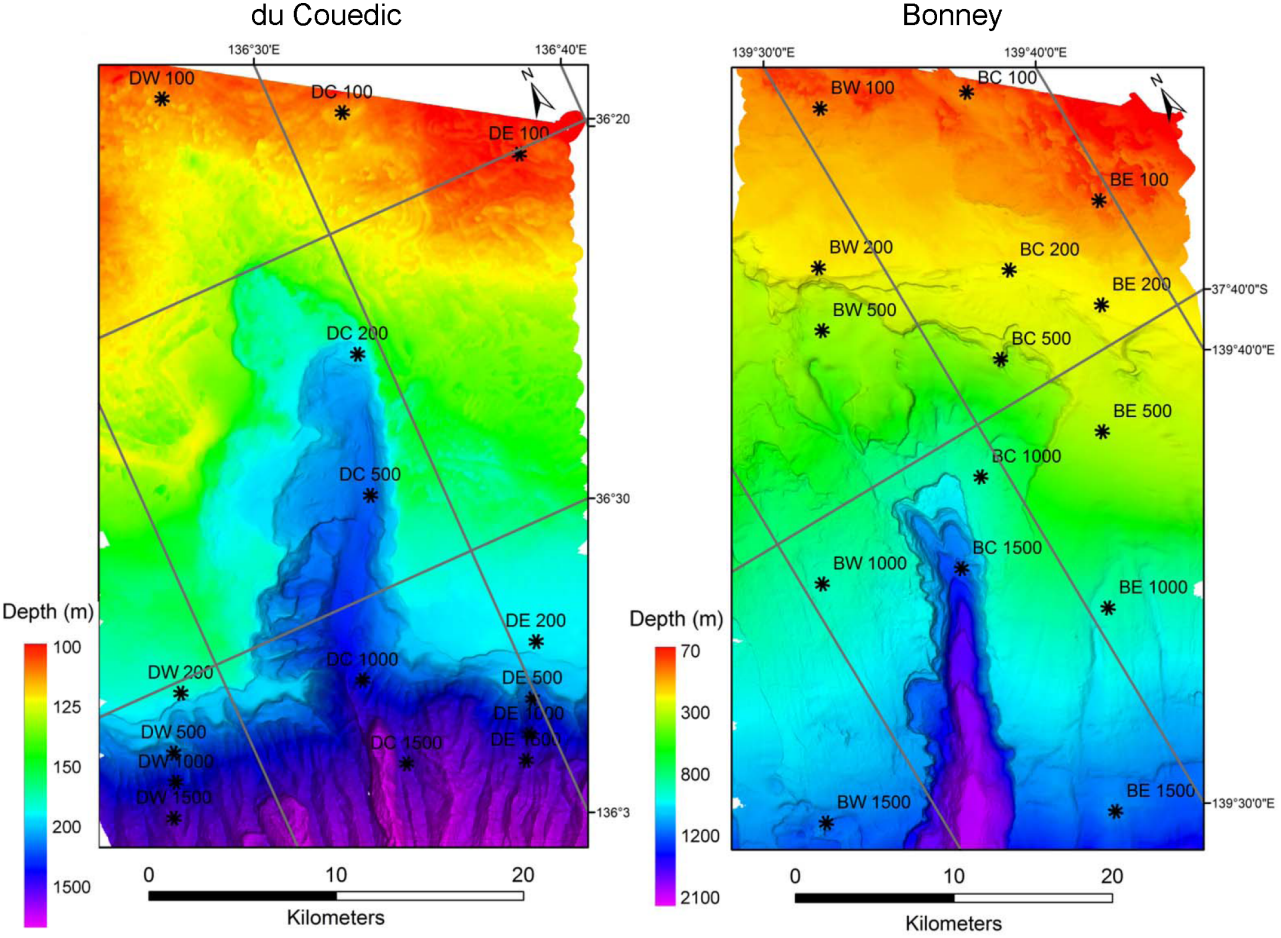
Multibeam acoustic images of the du Couedic and Bonney Canyon regions showing sampling stations coded by region (du Couedic or Bonney), transect (West, Centre or East) and depth (100, 200, 500, 1000 or 1500 m). The West and East stations are outside the canyons while the Centre stations run through the canyon axes. Note different scales of view. Macrofauna were collected at all but DW 1500, DE 1000 and DE 1500.

Du Couedic Canyon is a large and complex shelf-incising canyon that comprises one of the 95 such canyons on the Australian continental margin (Figs 1 and 2). Bonney Canyon is one of the common slope-confined canyons of which there are 618 surrounding mainland Australia [17]. Du Couedic Canyon is 300 km west of Bonney Canyon and incises the shelf for about 20 km, with a head wall at 200 m depth (Table 1). Bonney Canyon begins at 500 m depth with a more abrupt head wall at 800 m. Du Couedic Canyon is more complex in morphology and less isolated from other canyons than Bonney Canyon.

**Table 1:** Physical features of du Couedic and Bonney Canyons.

Feature	du Couedic	Bonney
Canyon type	Shelf-incising	Slope-confined
Shelf incision length (km)	20	0
No. branches	25	1
Distance to adjacent canyons (km)	0.2	10.6
Head location	36.3916°S, 136.4859°E	37.642°S, 139.5383°E
Head depth (m)	200	500
Max. width (km)	≈5	≈3
Max. depth (m)	>5000	>3000
Sidewall gradient	>1:1	>1:1
Canyon floor	Channels, chutes and turbidity current holes from slumping events	Terraced, indicating erosion and slumping

Data from [14; 17; 26; 29].

### Field methods

Collections of macrofauna, bottom water and surface sediment were made during voyage SS02/2008 aboard the Australian National Facility RV *Southern Surveyor* over 4–26 February 2008. None of the sites sampled were within the Commonwealth Marine Reserves Network or was under any other protection status at the time of sampling. This research and the collection of specimens was conducted by the South Australian Government under the direction of Dr. David Currie (second author, scientist with the South Australian Research and Development Institute, SARDI). In South Australia, the legislation covering animal welfare is the Prevention of Cruelty to Animals Act 1985 and Prevention of Cruelty to Animals Regulations 2000 (http://www.legislation.sa.gov.au/). Authority for these collections was covered by Schedule 1 of the Fisheries Management Act 2007, Section 115 (http://www.legislation.sa.gov.au/) which permits staff members of SARDI to “take any species of fish using any type of device, except explosives, from any waters of the state”. Under this act, "fish" refers to any aquatic animal other than an aquatic bird, an aquatic mammal, a reptile or an amphibian. This act applies to all commonwealth waters adjacent to the state that are within the Australian fishing zone. All specimens collected were invertebrates and none are listed under the Environment Protection and Biodiversity Conservation Act 1999 (EPBC Act) as threatened, endangered or rare. In addition, none are listed by the Convention on Trade in Endangered Species (CITES) as threatened by international trade.

The sampling design was balanced with five depths sampled in each of three transects in each of the du Couedic and Bonney regions (Figs 1 and 2). The east and west transects were outside the canyons (termed “exterior” or “outside”) while the Centre transect ran through the canyon axes (termed “interior” or “inside”). The depths were targeted to the shelf (100 m and 200 m), the upper slope (500 m) and lower slope (1000 m and 1500 m). The 100–200 m stations were in the coastal and shelf-edge South Australian Current, the 500 m stations were in the westward flowing Flinders Current and the 1000–1500 m stations were in the Antarctic Intermediate water mass [18; 26; 29]. These stations reside within their associated water masses for most of the year [18]. Equipment failure at the deepest stations in the du Couedic region, caused by the steep topography meant that no samples were taken at 1000 m depth to the east of the canyon axis and at 1500 m depth to the west and east of the canyon axis. Accordingly, 12 stations were sampled for the du Couedic region and 15 for the Bonney region. Sampling for bottom water, surface sediment and macrofauna occurred over 7–21 February 2008. Fish and megafauna were also collected and their patterns have been reported elsewhere [26; 29]. Macrofauna were collected by a 0.1 m^2^ Smith-McIntyre grab and organisms ≥1 mm were separated from the sediment by sieving, fixed in 4% formalin-seawater and transferred to the laboratory. Fragments of megafauna captured by the grab, such as sponges, were also retained. The 1 mm sieve mesh size was chosen in order to follow the same sampling protocol of [34] on the adjacent Great Australian Bight. Limited ship time for collection of water, sediment, fish, megafauna and macrofauna and frequent grab malfunction at the deeper depths prevented replication for macrofauna at the 27 stations. Thus, in this study, the terms “station” and “sample” are synonymous.

Twenty-six bottom water and surface sediment characteristics were measured along with gear performance and bathymetry at all stations except DE 1000, DE 1500 and DW 1500. The bottom water variables were temperature, salinity, fluorescence, PAR, oxygen, silicate, nitrate and phosphate. Water temperature, salinity and pressure were recorded at each sampling site using a Seabird SBE911 CTD fitted with modular sensors for dissolved oxygen (Aanderaa Optode 3975) and fluorescence (Chelsea AQUAtracka). All of these instruments were attached to the vessel’s 24-bottle rosette frame and lowered to within 20 m of the seabed during each cast. Nitrate, phosphate and silicate levels of the near-bed water were determined from laboratory analyses of water collected in a Niskin bottle. Surface sediment was collected from a second grab. Variables were proportion of particles <63μm, sorting coefficient, nitrogen and sulphur content. Carbon was also analyzed but due to equipment failure, the results could not be used. Bathymetry and geographic variables were pressure, depth, latitude, longitude and categorical descriptors of region and canyon definition. The gear performance was measured as weight of sediment collected by the macrofaunal grab. Further collection and analytical methods for the environmental data are described in [26; 29].

### Laboratory methods

Macrofauna were extracted from the residual sediment, weighed and preserved in 70% ethanol. All specimens were in good condition at the time of identification and there was no shell erosion evident for the molluscs. Specimens were identified to named or putative species using current taxonomic literature and checked against the World Register of Marine Species (http://www.marinespecies.org/). Taxa and their identifiers were: crustaceans, E. H., V. T. and S. C.; sponges, S. S.; all others, K. C. Nearly 2200 specimens were collected at the 27 stations and identified to 531 species or apparent species. Of these, 531 could be assigned to a phylum, 513 to class, 488 to order, 455 to family and 307 to genus. Nematodes and foraminiferans were occasionally caught but as they are generally considered as meiofauna they were excluded from the analysis. Feeding characteristics of dominant organisms were determined from [35–43].

Abundance was determined as the number of individuals with heads present if solitary. Abundance of colonial organisms required an arbitrary assignment of an abundance of 1 if present. Biomass was quantified by damp-dry weight after 5 minutes air drying on two layers of paper towels. Weight <0.01 g was scored as 0.01 g. Weight of shelled organisms was converted to shell-free wet-weight using the conversions of [44].

All fauna are stored at the South Australian Museum. The data are stored at the South Australian Research and Development Institute, the South Australian Museum and the Canadian Museum of Nature and are also available as S1 Data.

### Data analyses

The community composition matrix of 531 species x 27 stations was composed of abundances for the 442 solitary organisms (annelids, arthropods, echinoderms, molluscs and others) and biomass for the 91 colonial organisms (sponges, hydroids and bryozoans). For describing community structure, biomass was considered a better way to quantify the colonial organisms than an assignment of presence, which would have under-represented large colonial organisms. The DIVERSE routine of Primer v6 [45] was applied to determine species richness and expected species richness richness [46; 47], Shannon-Weaver diversity (log_e_) [48], Simpson’s [49] and Pielou’s [50] evenness and taxonomic distinctness [51]. Abundance, biomass and two diversity indices (species richness, which is sample size dependent and taxonomic distinctness, which is not [51; 52]) were mapped with ArcGIS 10.1 with bins defined by the Jenks Iterative method which minimizes within-class differences and maximizes between-class differences [53]. Estimated species richness was computed using the Chao 1 index [54; 55] using the program EstimateS [56]. Samples at each depth interval were averaged (n = 3, except n = 2 at D 1000). Values were abundance for solitary organisms and biomass for colonial organisms.

For analysis of dissimilarities among stations, the data were square root transformed to reduce the overwhelming effect of large values and then standardized by totals to remove the effect of the mixed quantification. Dissimilarities were calculated by the Bray-Curtis method [57] and visualized by non-metric multidimensional scaling (MDS) [58][59]. PERMANOVA [60] was applied to the transformed and standardized Bray-Curtis dissimilarities to test the hypotheses that the macrofauna (H1) are zoned by water mass but differ (H2) between the two canyons and (H3) between the interior and exterior of each canyon. The canyon interior was defined in two ways: (1) a broader definition as all samples taken along the central axis of each canyon, regardless of depth (transects DC and BC in Fig 2) and (2) a narrower definition as only those central axis stations that were in the distinctly visible interior of the canyon as shown in Fig 2, which was ≥200 m depth for du Couedic Canyon and ≥500 m depth for Bonney Canyon. These two definitions were termed “central canyon axis” and “topographically distinct interior”. The design was crossed with each factor fixed. The maximum number of label permutations for the calculation of pseudo-*F* was 999. Differences were considered significant at p<0.05. The unbalanced number of samples caused by equipment failure was addressed by using the Type III sum of squares partial analysis as recommended by Anderson [60]. Individual species contributions to community patterns were examined by canonical analysis of principal coordinates (CAP) [60] and the similarity percentages (SIMPER) routine in Primer 6 [45].

Environmental variables were examined for normality and then transformed by square root (if a percentage) or natural log otherwise, followed by normalization. Correlated variables (Pearson correlation ≥0.8) were reduced to a single variable acting as proxy for the other(s), leaving 13 uncorrelated variables. Distance-based linear models (DISTLM) and distance-based redundancy analysis (db RDA) [60] were applied to find linear combinations of these variables that explained the greatest variation in community composition. Model selection was step-wise with adjusted R^2^. Vector overlay was of multiple partial correlations of the centred predictor variables with the db RDA axes.

## Results

### Environmental characteristics

The two canyons resembled each other in bottom water characteristics at the time of sampling (S1 Table). Characteristics inside the canyons (C) were comparable to the outside (west (W) and east (E)) at the same depth. Bottom water temperature varied from 12.6°C on the shelf to 2.7°C at 1500 m. Oxygen, fluorescence and PAR also declined with depth. Salinity varied little, with the highest salinity (35 psu) occurring at 200 m in both regions. Silicate, nitrate and phosphate increased with depth to a maximum of 82, 36 and 3 μMl^-1^ respectively.

Mud content of the surface sediments generally increased with depth but the two upper slope sites within the canyon axes (BC 500 and DC 500) broke this trend by having very coarse, poorly sorted sediments with <6% mud content (S2 Table). Sediment nitrogen content increased with depth to a maximum of 0.19% but the two upper slope sites in the canyon axes had lower levels than at their same depth counterparts outside the canyons. Sediment sulphur showed no clear trend with depth. Gear performance was variable and was not clearly related to mud content. However it did decline at greater depth, capturing a smaller volume or in some cases none at all.

### Community structure

#### Regional characteristics

Of the 531 species of macrofauna in the analysis, 319 (60%) were found in the du Couedic region and 343 (65%) in the Bonney region (Table 2). Sampling effort was less in du Couedic than Bonney regions, however, with 12 and 15 stations, respectively and each station with one sample. The two regions had 131 (25%) species in common. In each region, most of the species were captured at only one station (71 and 66% for du Couedic and Bonney regions, respectively) and only 16 species were present at >3 stations. The maximum frequency of occurrence for any species was 7 of the 12 stations in the du Couedic region and 9 of the 15 stations in the Bonney region, equivalent to 58–60% of the stations. There were 188 species that were unique to the du Couedic region (59% of the total) while there were 212 unique to the Bonney region (62% of the total). Of the 319 unique or non-unique species captured at du Couedic stations, 223 were restricted to the shelf, 72 were restricted to the slope and 24 occurred on both the shelf and slope. This amounted to 40, 23 and 7% of the total captured. In the Bonney region, 255 and 64 were restricted to the shelf and slope, respectively and 24 occurred on both, giving a proportion of 74, 19 and 7%, respectively.

**Table 2:** Species characteristics of the du Couedic and Bonney macrofaunal samples.

Species characteristic	du Couedic		Bonney	
	No.	%	No.	%
Total present	319	60	343	65
Present at only 1 station	226	71	225	66
Present at >3 stations	16	5	16	5
Max. frequency of occurrence	7	58	9	60
Unique to the region	188	59	212	62
Restricted to the shelf	223	70	255	74
Restricted to the slope	72	23	64	19
On both the shelf and slope	24	7	24	7

There were 12 du Couedic and 15 Bonney stations with 531 species collected. Shelf stations are at 100–200 m); slope stations are at 500–1500 m).

**Fig 3:**
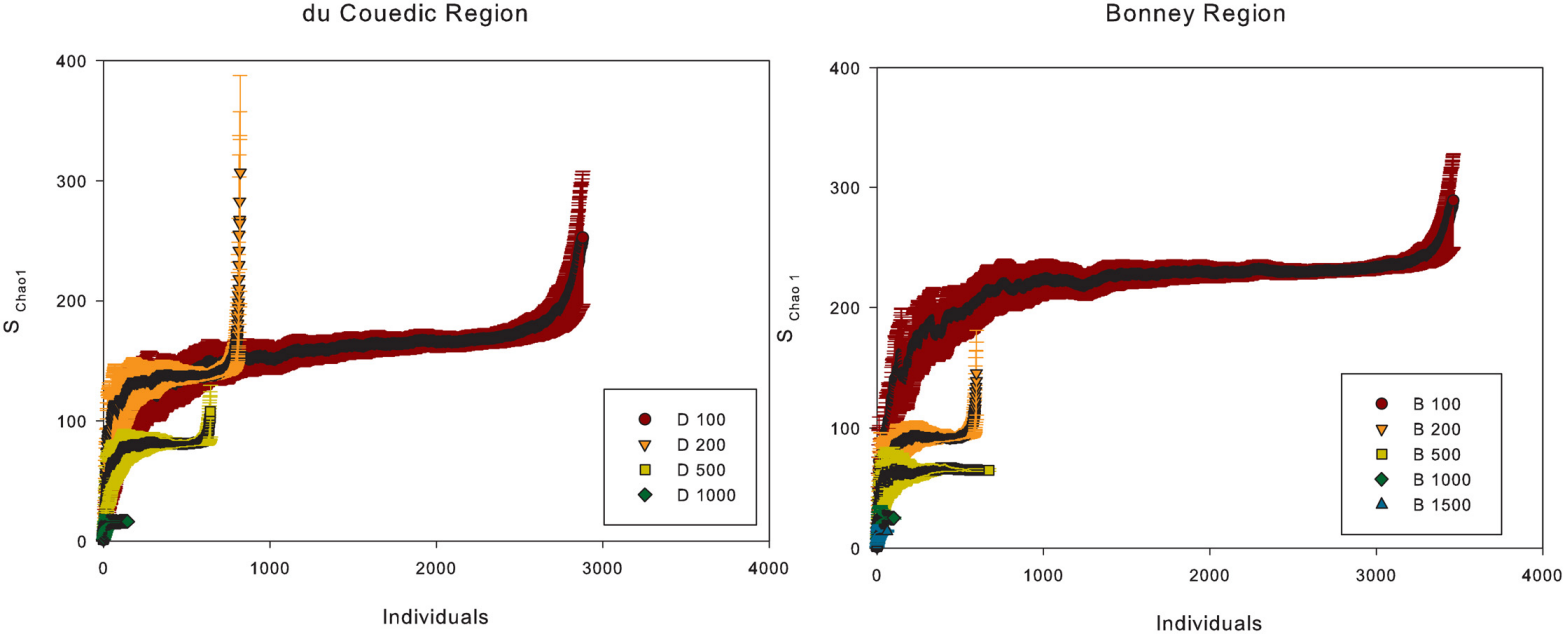
Rarefaction curves of estimated species richness based on the Chao 1 index for the du Couedic and Bonney regions at each depth interval. The x axis represents the number of individuals randomly subsampled with replacement from within the sample. The y axis is the expected mean number of species using the Chao 1 index.

Estimated species richness within each depth interval, based on rarefaction curves of the Chao 1 index, is compared for the du Couedic and Bonney regions, respectively (Fig 3). Comparing D 100–1000 with B 100–1000 at the n = 16 runs for the least species-rich location, mean S _Chao 1_ was 92.2 ± 30.2 vs 126.4 ± 31.8 at 100 m, 115.5 ± 25.0 vs 77.4 ± 14.4 at 200 m, 70.0 ± 13.9 vs 59.6 ± 13.3 at 500 m and 16.0 ± 0.6 vs 25.0 ± 0.9 at 1000 m for the du Couedic vs Bonney regions, respectively. The D 100 and D 200 m curves overlapped at their asymptote while the others did not. The D 100–500 and B 100–200 curves rose rapidly in mean and SD at the upper ends of the curves while the others did not.

#### Station characteristics

**Fig 4:**
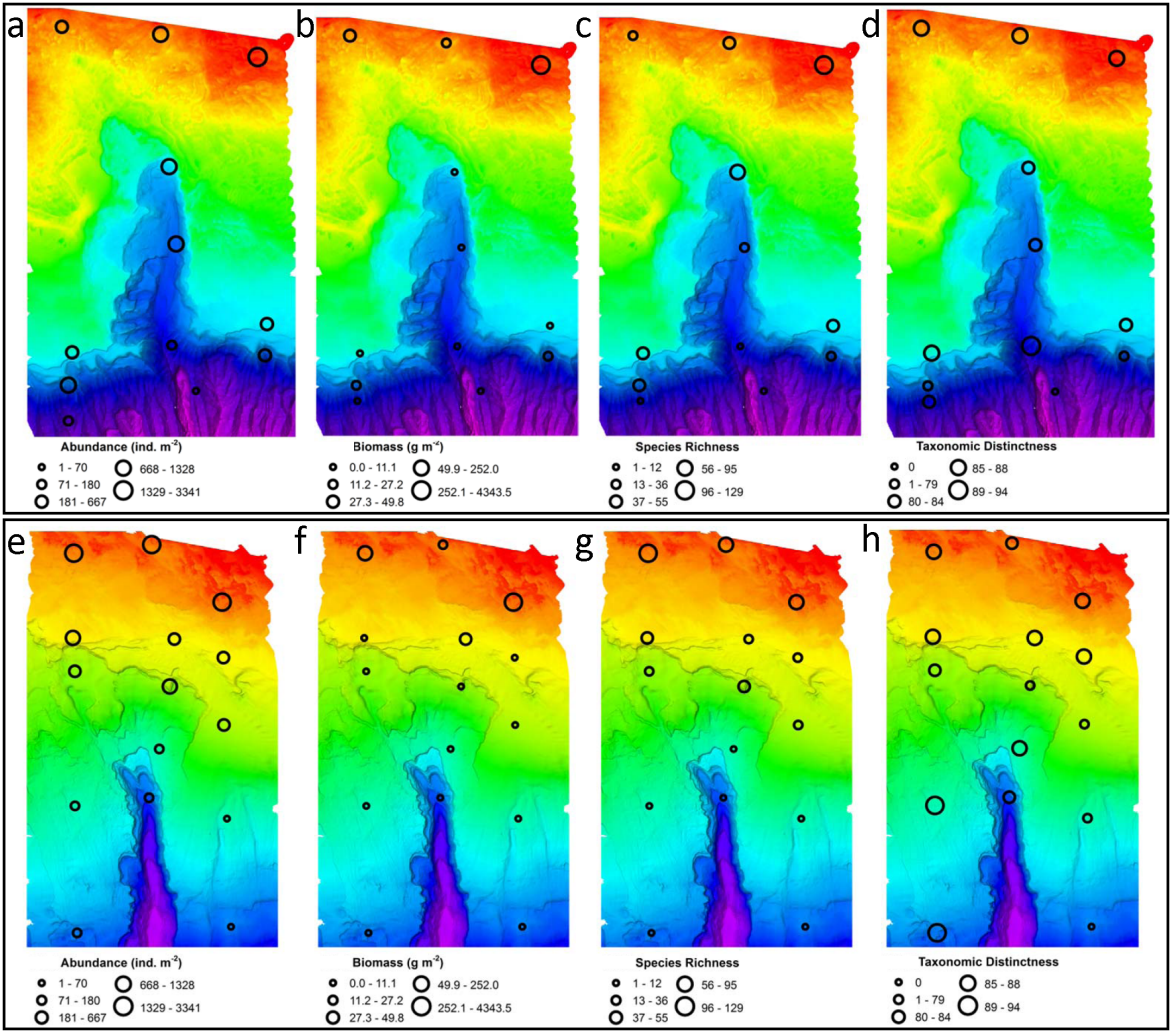
Abundance (a and e), biomass (b and f), species richness (c and g) and taxonomic distinctness (d and h) at each station in Fig 2. Upper panel: du Couedic region; lower panel: Bonney region. The same scale is used for the same variable in each region.

Maximum abundance (3341 and 3164 ind m^-2^) and biomass (4343.5 and 2516.0 g m^-2^) occurred outside the canyons at 100 m in the du Couedic and Bonney regions, respectively (Table 3). Both abundance and biomass declined with depth to ≤10 ind m^-2^ and a biomass of ≤0.1 g m^-2^ (Table 3, Fig 4). The proportion of biomass to abundance for the solitary organisms (i.e., annelids, molluscs, arthropods, echinoderms and other solitary species) did not decline with depth. Highest proportions were at DE 500 (0.051) and BW 1500 (0.040) for the du Couedic and Bonney regions respectively. Species richness of the total macrofauna was highest at DE 100 (121 species) and BW 100 (129 species) and declined with depth to a single species at DC 1500 and BE 1500. The expected number of species from a sample of 400 individuals was highest in the du Couedic region at DC 200 (67.88) and in the Bonney region at BW 100 (94.42). Values declined with depth particularly deeper than 500 m. Shannon-Weaver diversity also declined with depth, ranging from 3.98 at DC 100 to 1.47 at DC 1000 in the du Couedic region and 4.02 at BW 100 to 1.79 at BE 1000 in the Bonney region. Community structure generally remained evenly distributed and taxonomically distinct with depth, with most values >0.8 for Pielou evenness, >0.9 for Simpson evenness and >0.8 for taxonomic distinctness. The upper reaches of both canyons at 500 m (DC 500) and (BC 500) showed lower Shannon-Weaver diversity due to higher dominance (less evenness) in the central canyon axes than outside the canyons to west or east at this depth (3.37–2.59–3.00 at du Couedic and 2.82–2.49–3.19 at Bonney for Shannon-Weaver index, 0.90–0.78–0.93 and 0.90–0.69–0.96 for Pielou index and 0.95–0.89–0.95 and 0.92–0.78–0.95 for Simpson index, for W-C-E respectively). Taxonomic distinctness was high (>75.5) at all stations with more than one species and highest where a few distantly related taxa occurred in the deep stations (90.67 at DC 1000 and 94.00 at BW 1500).

**Table 3:** Abundance, biomass, ratio of solitary species biomass to abundance, species richness, expected species richness and four indices of diversity for the macrofauna collected at each of the stations in Fig 2.

Station	N	B	B/N	S	ES_400_	H’	J’	1-λ’	Δ*
DW 100	437	45.9	0.013	30	26.00	3.09	0.91	0.95	86.73
DC 100	807	27.2	0.006	47	40.98	3.31	0.86	0.94	84.77
DE 100	3341	4343.5	0.023	119	61.68	3.13	0.65	0.90	86.96
DW 200	518	11.1	0.005	40	33.00	3.31	0.90	0.96	86.18
DC 200	1328	7.4	0.005	77	67.88	3.98	0.92	0.98	83.83
DE 200	667	7.4	0.009	54	47.60	3.71	0.93	0.97	84.03
DW 500	851	17.6	0.018	41	40.69	3.34	0.90	0.95	78.47
DC 500	866	8.2	0.009	27	21.00	2.51	0.76	0.88	79.59
DE 500	401	20.6	0.051	25	24.00	3.00	0.93	0.94	77.37
DW 1000	180	8.3	0.046	10	10.00	2.03	0.88	0.84	80.42
DC 1000	140	2.0	0.014	6	6.00	1.47	0.82	0.70	90.67
DC 1500	1	0.1	0	1	0	0			0
BW 100	3164	252.0	0.009	129	94.42	4.02	0.83	0.95	84.97
BC 100	2803	13.1	0.005	72	62.17	3.53	0.82	0.94	83.35
BE 100	2000	2516.0	0.011	95	66.19	3.29	0.72	0.91	87.21
BW 200	877	8.4	0.009	41	33.97	3.11	0.84	0.93	85.46
BC 200	483	49.8	0.024	35	33.00	3.30	0.93	0.95	87.10
BE 200	474	9.6	0.020	37	33.00	3.33	0.92	0.96	86.06
BW 500	420	1.4	0.003	23	23.00	2.82	0.90	0.92	81.13
BC 500	1190	3.4	0.003	37	36.61	2.46	0.68	0.77	76.86
BE 500	450	2.1	0.005	28	28.00	3.19	0.96	0.95	78.56
BW 1000	120	2.9	0.024	10	10.00	2.21	0.96	0.88	89.42
BC 1000	150	2.8	0.018	12	12.00	2.44	0.98	0.91	88.20
BE 1000	70	0.3	0.004	6	6.00	1.79	1.00	0.85	75.56
BW 1500	91	3.7	0.040	9	8.00	2.09	0.95	0.89	92.28
BC 1500	111	0.4	0.003	7	6.00	1.65	0.85	0.79	77.83
BE 1500	10	0.1	0	1	1.00	0		0	0

N: Abundance (ind m^-2^); B: Biomass (g m^-2^); S: Species richness; ES_400_: Expected number of species in a sample of 400 individuals; H’: Shannon-Weaver index; J’: Pielou index; 1-λ’: Simpson index with individuals chosen without replacement; Δ*: Taxonomic distinctness. Colonial organisms are scored by presence for abundance and by biomass for all others.

**Fig 5:**
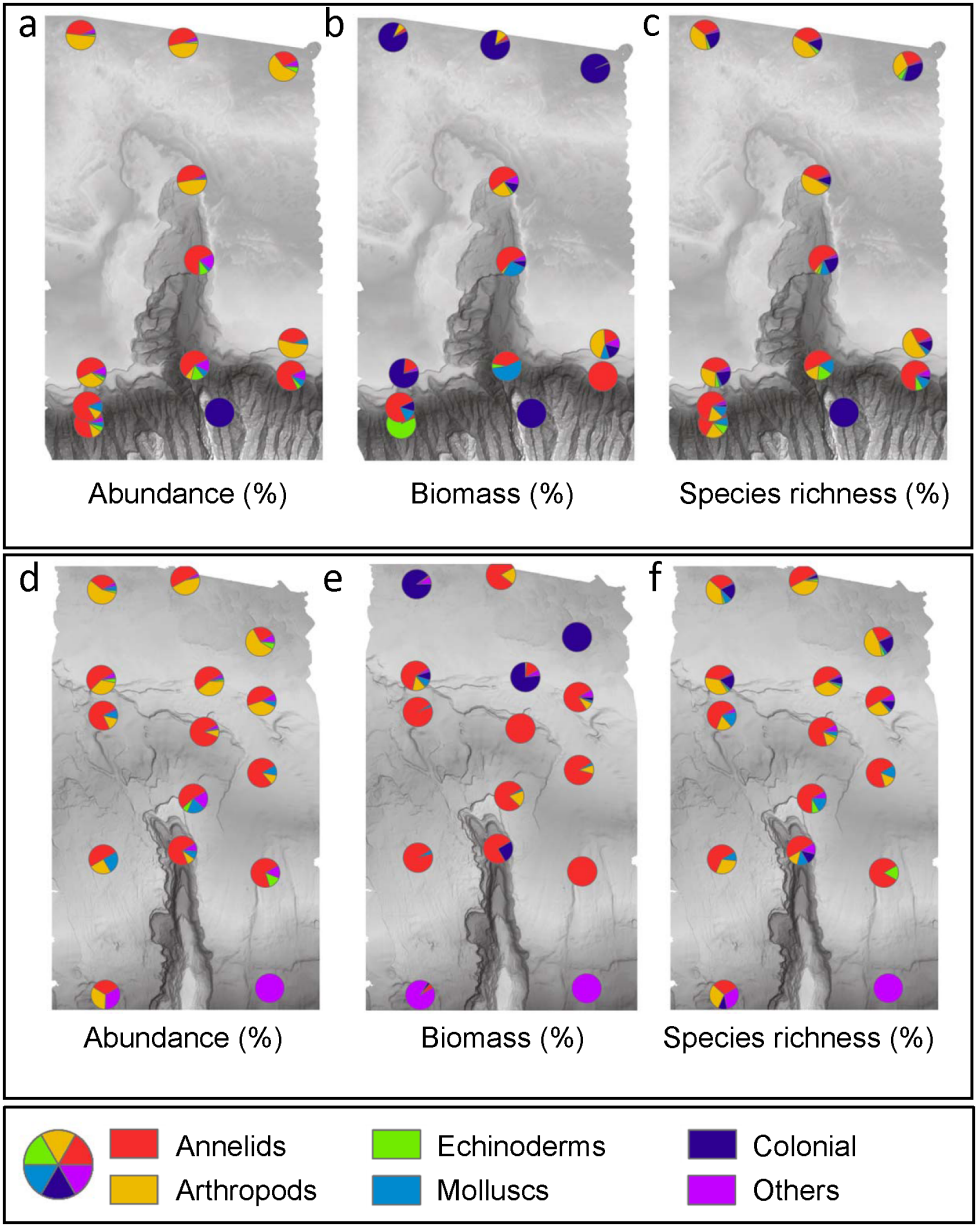
Distribution of abundance (a and d), biomass (b and e) and species richness (c and f) among the major taxa, shown as pie charts for each station in Fig 2. Upper panel: du Couedic region; lower panel: Bonney region. Major taxa and their colour codes are, annelids (red); arthropods (yellow); echinoderms (green); molluscs (turquoise); colonial organisms (mainly sponges) (navy); other solitary organisms (e.g., an enteropneust at BW 1500 and a nemertean at BW 1500) (purple).

Among the six major taxa, annelids and arthropods accounted for the largest proportion of abundance on the shelf (up to 94.5% at 100–200 m) while on the slope (500–1500 m), the arthropods (primarily peracarids) declined in proportion, leaving the annelids (primarily polychaetes with some oligochaetes) as the sole abundance dominants (up to 84.9%) at most stations (Fig 5a and 5d). The only case where another taxon was the dominant was when abundance was very low, giving a few species a large proportion of the abundance (e.g. a calcareous sponge at DC1500 and a nemertean at BE1500).

Colonial organisms (predominantly sponges) dominated the biomass on the shelf (up to 99%), giving a total biomass ranging from 7.4 g to 4.3 kg m^-2^ at 100–200 m (Fig 5b and 5e). On the slope, biomass was low (0–20.6 g m^-2^) and was primarily accounted for by annelids. Molluscs accounted for >25% of total biomass in du Couedic Canyon at 500 and 1000 m but <15% elsewhere. At 1500 m, a calcareous sponge, an enteropneust and a nemertean dominated the biomass at DC, BW and BE, respectively.

Most species on the shelf were colonial organisms, annelids or arthropods (Fig 5c and 5f). On the slope, annelids accounted for 30–83% of the species content of a sample. Molluscs and echinoderms were present throughout the range of depths with up to 10 species on the shelf and up to 5 species on the slope in any one sample. Most of these major taxa accounted for 3% or less of the Australian species richness as defined by [61]. However, the 173 species of annelids accounted for 11% of the known Australian annelids. Species richness was split among major groups at most stations except for DC 1500 and BE 1500 where a calcareous sponge and a nemertean were the sole organisms found.

**Fig 6:**
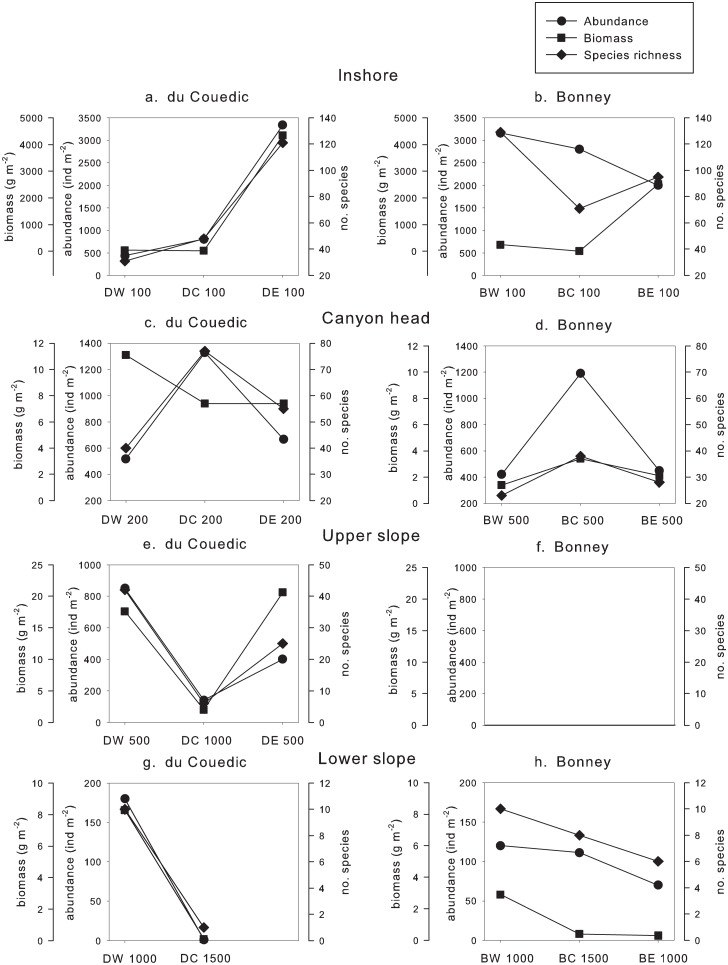
Cross-canyon abundance (circle), biomass (square) and species richness (diamond) inshore (a and b), at the canyon head (c and d), on the upper slope (e and f) and on the lower slope (g and h) inside (C) and outside (to west (W) and east (E)) of du Couedic (D) and Bonney (B) Canyons. “Upper and lower slope” are relative terms spanning the range of this study. There were no data for (f) or for DE 1000. Distances between stations are shown in Fig 2. Station codes are as in Fig 2.

When the samples were compared as cross-canyon transects (Fig 6), different patterns emerged. Inshore, at 100 m, there were more than two orders of magnitude increases in biomass to the east (E) of the central canyon axis (C) and the western transect (W) in both the du Couedic (27.2 to 4343.5 g m^-2^) and Bonney (13.1 to 2516.0 g m^-2^) regions (Fig 6a and 6b). A near order of magnitude increase in abundance (437 to 3341 ind m^-2^) and species richness (31 to 121 species) corresponded with the biomass increase in the du Couedic region but not in the Bonney region, where abundance and species richness instead declined from W to E (3164 to 2000 ind m^-2^ and 129 to 95 species).

The canyon heads (at 200 m depth for du Couedic and 500 m for Bonney) had ≥2x the abundance in the centre (C) than outside the canyons to W or E (Fig 6c and 6d). At the du Couedic Canyon head (DC 200), abundance was 1328 ind m^-2^ compared to 518 ind m^-2^ at DW 200 and 667 ind m^-2^ at DE 200 (Fig 6c). At Bonney Canyon, abundance at the head (BC 500) was 1190 ind m^-2^ compared to 420 ind m^-2^ at BW 500 and 450 ind m^-2^ at BE 500 (Fig 6d). Species richness at the du Couedic Canyon head (77 species at DC 200) was nearly double that to either side (40 species at DW 200 and 55 species at DE 200). At the Bonney Canyon head, species richness was 1.5x greater (38 species at BC 500) than to either side of the head (23 species at BW 500 and 28 species at BE 500). Biomass was less at the du Couedic Canyon head (7.4 g m^-2^) than to W (11.1 g m^-2^) but the same as to E (7.4 g m^2^). At the Bonney Canyon head, biomass followed the pattern of abundance and species richness with >1.5x higher biomass at BC 500 (3.4 g m^-2^) than at BW 500 (1.4 g m^-2^ and BE 500 (2.1 g m^-2^).

On the upper slope, depth doubled from 500 m at W and E to 1000 m in the centre (C) of du Couedic Canyon (Fig 2). Abundance inside the canyon dropped to less than half of that outside (140 ind m^-2^ at C compared to 851 and 401 ind m^-2^ at W and E, respectively). Biomass inside the canyon was around 10% of that outside (2.0 g m^-2^ at C compared to 17.6 and 20.6 g m^-2^ at W and E). Species richness was <25% of that outside the canyon (42 and 25 species at W and E, respectively compared to 6 species at C) (Fig 6e). At Bonney, there was no comparable cross-canyon transect on the upper slope (Figs 2 and 6f).

On the lower slope (1000 m depth W and E of the canyon axis and 1500 m in the centre), abundance, biomass and species richness were near zero inside du Couedic Canyon (1 ind m^-2^, 0.1 g m^-2^ and 1 species at DC 1500) but substantially higher outside and to the west (180 ind m^-2^, 8.3 g m^-2^ and 10 species at DW 1000) (Fig 6g). These variables were also lower inside Bonney Canyon though not as substantially (111 vs 120 ind m^-2^, 0.4 vs 2.9 g m^-2^ and 8 vs 10 species at BC 1500 compared to BW 1000) (Fig 6h). These variables were lower still at E for Bonney (70 ind m^-2^, 0.3 g m^-2^ and 6 species) but unknown for du Couedic due to equipment failure at DE 1000.

Further sampling down-slope (≥1500 m depth) was successful outside of Bonney Canyon at BE and BW but not outside of du Couedic Canyon. Comparative sampling within the canyons was attempted at 2000 m but did not succeed. Abundance, biomass and species richness at BW 1500 and BE 1500 were respectively 91 and 10 ind m^-2^, 3.7 and <0.01 g m^-2^ and 10 and 1 species (Fig 4).

#### Hypothesis tests

**Fig 7:**
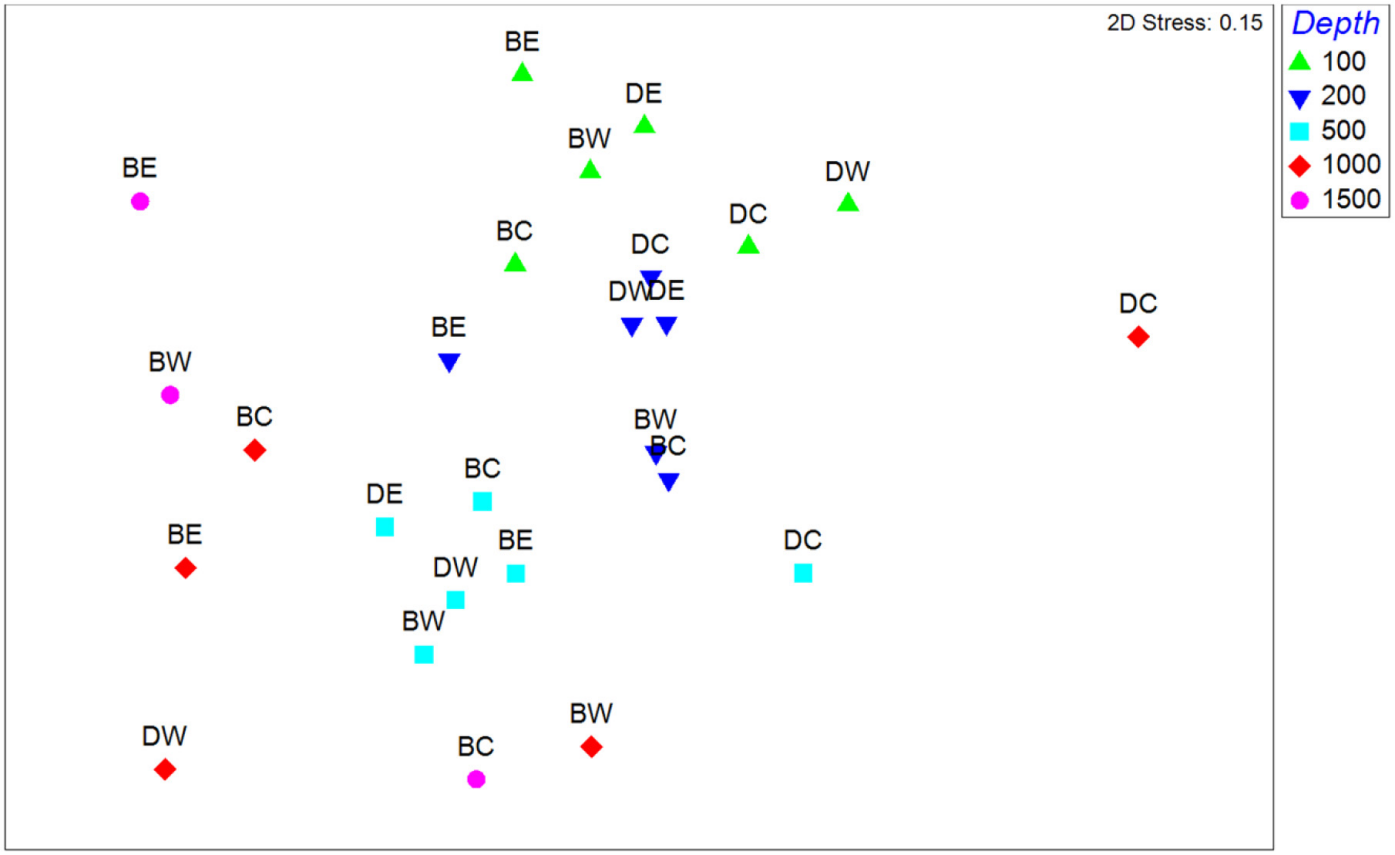
Unconstrained non-metric multidimensional scaling plot of station dissimilarities based on community composition. DC 1500 is not shown because its dissimilarity to other stations was so high that it prevented resolution of the relationships of the other stations. Station codes are as in Fig 2.

Community composition changed significantly with water mass (p = 0.001 and 0.003) when the canyon interiors were defined by the “central canyon axis” and “topographically distinct interior”, respectively (H1) (Table 4). The regions were close to being significantly different (p = 0.06 and 0.07, respectively) (H2). There was no significant difference between the interior and exterior of the canyons by either definition of canyon interior (H3). Interactions were not significant. The change in community composition with depth (and therefore water mass since the three water masses stratify by depth) is evident in the unconstrained multidimensional scaling (MDS) ordination (Fig 7). Shallower (100–500 m) stations grouped more tightly than the deep (1000–1500 m stations). There was high resemblance between the station at the head of du Couedic Canyon (DC 200) and the stations at the same depth to west and east (DW 200 and DE 200). At 500 and 1000 m, though, community composition in the centre of du Couedic Canyon was quite different from outside. Community composition at the head of Bonney Canyon (BC 500) was not as distinctive relative to the canyon exterior (BW 500 and BE 500) as at du Couedic Canyon. There was not a consistent distinction of du Couedic from Bonney samples (H2) although some same-region samples showed close similarity within the same depth (e.g., du Couedic samples at 200 m). Similarly, samples from the interior (H3) (DC and BC) of the canyons did not separate from exterior samples (DW, DE, BW and BE).

**Table 4:** PERMANOVA of community composition with the two contours (Inside (I) vs Outside (O)), three water masses (WM) and two regions (R) fixed factors.

	Central canyon axis				Topographically distinct interior			
Source	*df*	*MS*	*F*	*P (perm)*	*df*	*MS*	*F*	*P (perm)*
I-O	1	4805.3	1.25	0.19	1	3367.8	0.89	0.65
WM	2	8367.5	2.18	0.001**	2	6182.9	1.63	0.003**
R	1	5547.3	1.45	0.06	1	5557.5	1.46	0.07
I-O x WM	2	4234.6	1.10	0.33	2	4189.4	1.10	0.31
I-O x R	1	3251.0	0.85	0.72	1	3388.5	0.89	0.62
WM x R	2	4398.9	1.15	0.21	2	4336.4	1.14	0.24
I-O x WM x R	2	3482.3	0.91	0.68	1	3929.6	1.04	0.44
Residual	15	3837.0			16	3795.7		
Total	26				26			

Contour compares the interior to the exterior of each canyon. Two definitions of interior were used: all samples taken within the central canyon axis and all samples taken within only the parts of the canyon that are topographically distinct in Fig 2 (central axis ≥200 m for du Couedic Canyon and ≥500 m for Bonney Canyon). Canyon exterior was all samples that were not defined as interior. The three water masses are the South Australian current at 100–200 m, Flinders current at 500 m and Antarctic Intermediate Water at 1000–1500 m. The two regions are du Couedic and Bonney. Significant differences are indicated by

* (p<0.05) or

** (p<0.01).

**Fig 8:**
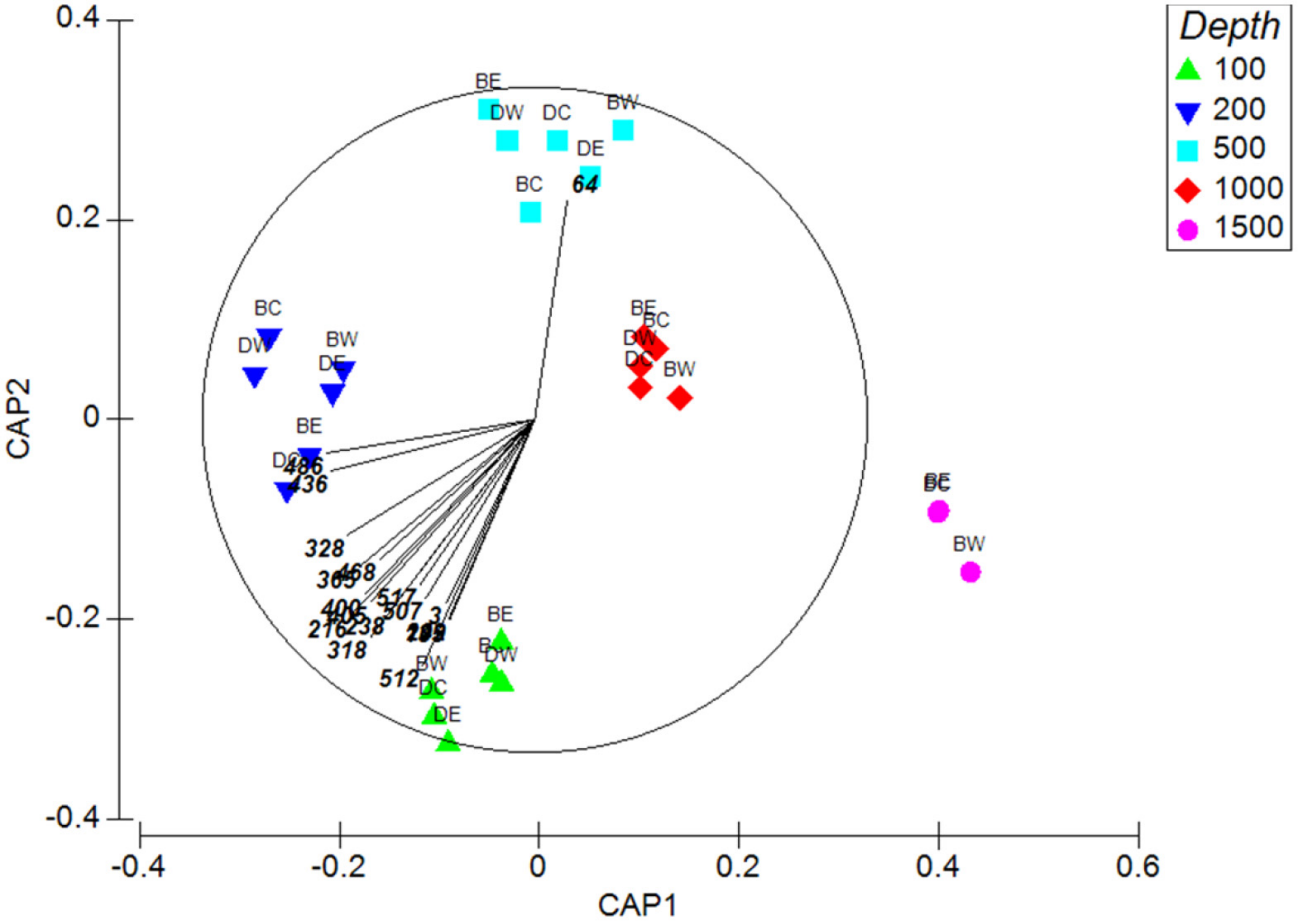
Constrained ordination of community composition by canonical analysis of principal coordinates (CAP) with a vector overlay of species having a Spearman rank correlation >0.6. Station codes are as in Fig 2. Species codes are listed in Table 5.

Canonical analysis of principal coordinates (CAP) showed the strong correlation of community composition with depth (H1) (*δ*_*1*_^*2*^ = 0.97 and *δ*_*2*_^*2*^ = 0.96), with the first axis separating the 100, 200, 1000 and 1500 m samples and the second axis separating the 100, 500 and 1500 m samples (Fig 8). Species most correlated with these axes (Spearman rank correlation >0.6) were the sponge *Phycopsis* sp., two bryozoans (*Cornuticella* sp. and an un-named species), five arthropods (the galatheid *Phylladiorhynchus pusillus*, the nebalian *Paranebalia* sp. and the amphipods *Leucothoe* sp., *Gammaropsis* sp. and *Ampelisca* sp.) and nine annelids (*Odontosyllis corruscans*, Syllinae sp. 1, *Amaeana* sp., Hesionidae sp., *Glycera* sp. 1, *Pareurythoe chilensis*, Sabellidae sp. 1, *Leptoecia* sp. and *Prionospio* sp. 1) (Table 5). The aplacophoran mollusc *Chaetoderma* sp. occurred only at 500 m. Many of these species were predators or suspension feeders.

**Table 5:** Species with Spearman rank correlations >0.6 on Fig 8 (CAP) ordered by association with increasing depth from 100 to 500 m.

Code	Species	Family	Phylum	Feeding type	Feeding depth
512	*Odontosyllis corruscans*	Syllidae	Annelida	P, D	S
49	*Leucothoe* sp.	Leucothoidae	Arthropoda	S	S
102	*Phylladiorhynchus pusillus*	Galatheidae	Arthropoda	P, D?	S
135	*Paranebalia* sp.	Paranebaliidae	Arthropoda	P, D, Sc	S
3	*Phycopsis* sp. I068	Axinellidae	Porifera	S	S
507	Syllinae sp. 1	Syllidae	Annelida	P, D	S
517	*Amaeana* sp.	Terebellidae	Annelida	D	S
318	*Cornuticella* sp.	Catenicellidae	Bryozoa	S	S
238	*Gammaropsis* sp.	Corophiidae	Arthropoda	S, D	S
216	*Ampelisca* sp.	Ampeliscidae	Arthropoda	S, D	S
405	Hesionidae sp.	Hesionidae	Annelida	P	S
400	*Glycera* sp. 1	Glyceridae	Annelida	P	S
365	*Pareurythoe chilensis*	Amphinomidae	Annelida	P, Sc	S
468	Sabellidae sp. 1	Sabellidae	Annelida	S	S
328	Branching bryozoan sp. 3		Bryozoa	S	S
436	*Leptoecia* sp.	Onuphidae	Annelida	D, Sc	S
486	*Prionospio* sp. 1	Spionidae	Annelida	S, D	S
64	*Chaetoderma* sp.	Chaetodermatidae	Mollusca	D	Su

Feeding types: D = deposit feeder; P = predator; S = suspension feeder; Sc = scavenger. Feeding depth: S = surface and near-surface; Su = subsurface.

Although overall community composition inside the topographically distinct canyons was not significantly different from outside (H3), some species showed contrasts in their distributions, as determined by similarity percentages (SIMPER) analysis (Table 6). For du Couedic Canyon, species that were more abundant inside than outside and collectively contributed to up to 30% of the average dissimilarity between contours were the polychaetes *Dodecaceria* sp., *Axiothella* sp., *Protodorvillea biarticulata*, *Prionospio* sp. 1 and *Lumbrineris* sp. 1 and a molpadid holothurian. Two polychaetes were more abundant outside than in (*Antiobactrum* sp. and *Aurospio* sp.). It required 25 species to collectively contribute to 50% of the average Bray-Curtis dissimilarity between du Couedic Canyon and its exterior while for Bonney Canyon, it required only 7 species. Three of these cumulatively contributed to up to 30% of the average dissimilarity. These were the polychaetes *Myrioglobula* sp., *Notomastus torquatus* and *Hemipodia* sp. 1, which were all more abundant inside than outside. Many of these species were surface deposit feeders or had mixed feeding modes.

**Table 6:** Species discriminating the interior from the exterior of du Couedic and Bonney Canyons that collectively contributed to 50% of the average Bray-Curtis dissimilarity of the group (SIMPER analysis).

Species	Family	Phylum	Feeding type	Feeding depth	Ave. abundance outside (ind. m^-2^)	Ave. abundance inside (ind. m^-2^)	Cumulative % contribution to ave. dissimilarity
du Couedic							
*Dodecaceria*?	Cirratulidae	Annelida	D	S	0	56.67	5.8
*Axiothella* sp.	Maldanidae	Annelida	D	Su	0	20	10.4
*Antiobactrum* sp.	Opheliidae	Annelida	D	Su	18	0	14.8
*Protodorvillea biarticulata*	Dorvilleidae	Annelida	D, Sc	S	0	40	19.0
Molpadida sp. 2		Echinodermata	S, D	S	0	30	22.1
*Aurospio* sp.	Spionidae	Annelida	S, D	S	8	0	25.1
*Prionospio* sp. 1	Spionidae	Annelida	S, D	S	6	43.3	28.0
*Lumbrineris* sp. 1	Lumbrineridae	Annelida	P, D	S, Su	12	26.7	30.45
*Linopherus* sp.	Amphinomidae	Annelida	P, D, Sc	S	28	0	32.4
Oligochaeta sp. 4		Annelida	D	S, Su	0	16.7	34.2
Loveniidae sp.	Loveniidae	Echinodermata	D	S, Su	0	6.7	35.7
*Hemipodia* sp.	Glyceridae	Annelida	P	S	2	16.7	37.2
*Meiodorvillea* sp.	Dorvilleidae	Annelida	D, Sc	S	0	13.3	38.6
*Melinnoides* sp.	Ampharetidae	Annelida	D	S	14	20	39.9
*Mooreonuphis* sp.	Onuphidae	Annelida	P, D, Sc	S	18	0	41.2
*Chaetozone* sp. 2	Cirratulidae	Annelida	D	S	12	0	42.3
*Marenzelleria*?	Spionidae	Annelida	S, D	S	6	10	43.3
*Aricidea pacifica*	Paraonidae	Annelida	D	S, Su	14	0	44.3
*Bathytanais fragilis*	Paratanaidae	Arthropoda	D?	S	0	13.3	45.2
*Isaeia*	Isaeidae	Arthropoda	D	S	0	13.3	46.2
Nemertea		Nemertea	P	S	6	10	47.1
*Chaetozone* sp. 1	Cirratulidae	Annelida	D	S	0	10	48.0
Oligochaeta sp. 5		Annelida	D	S, Su	4	6.7	49.0
*Gari* sp.	Psammobiidae	Mollusca	S	S	2	10	49.8
Corophiidae sp. 2	Corophiidae	Arthropoda	S, D	S	14	0	50.7
Bonney							
*Myrioglobula* sp.	Oweniidae	Annelida	S, D	S	6.7	180	13.3
*Notomastus torquatus*	Capitellidae	Annelida	D	S, Su	5	10	22.6
*Hemipodia* sp.	Glyceridae	Annelida	P	S	5	26.7	31.9
Nemertea		Nemertea	P	S	3.3	13.3	38.6
*Aricidea pacifica*	Paraonidae	Annelida	D	S, Su	13.3	13.3	43.5
Eulimidae	Eulimidae	Gastropoda	P	S	0	6.7	46.9
*Paraonis* sp.	Paraonidae	Annelida	D	S, Su	0	6.7	50.0

Canyon interior was defined as that part which was topographically distinct in Fig 2 (central axis ≥200 m for du Couedic Canyon and ≥500 m for Bonney Canyon). Canyon exterior was all samples to west or east of the canyons over the same depth range as the interior. Abundances are averaged across depth. Feeding designations: D = deposit feeder/grazer; P = predator/parasite; S = suspension feeder; Sc = scavenger. Feeding depth: S = surface and near-surface; Su = subsurface.

#### Relationship of community composition to environmental variables

Community composition varied significantly with latitude, bottom water salinity, fluorescence, PAR and oxygen and surface sediment mean grain size, sorting and nitrogen content (Table 7). Other variables were highly correlated (Pearson correlation >0.8) with some of these variables. Latitude correlated negatively with longitude and region; salinity correlated positively with temperature and negatively with silicate, nitrate and phosphate; oxygen correlated positively with temperature and depth and negatively with pressure, silicate, nitrate, phosphate and sediment mud content; and sediment nitrogen content correlated positively with mud content. Community composition did not significantly vary with grab weight, sediment sulphur content or canyon definition (“central canyon axis” or “topographically distinct interior”).

**Table 7:** Relationship between individual predictor variables and community composition determined by DISTLM.

Variable type	Predictor variable	Mean ± sd	Correlated variables	*SS* (trace)	*F*	*P (perm)*
Bottom water	Salinity (‰)	34.73 ± 0.25	Temperature, silicate, nitrate, phosphate	10652	2.59	0.0001**
	Fluorescence (μgl^-1^)	11.58 ± 1.90		10378	2.51	0.0001**
	PAR (volts)	0.12 ± 0.26		7565.1	1.78	0.002**
	Oxygen (μMl^-1^)	227.81 ± 32.20	Pressure, temperature, depth, mud content, silicate, nitrate, phosphate	9135.8	2.18	0.0001**
Surface sediment	Mean grain size (phi)	2.68 ± 1.37		7170.7	1.68	0.001**
	Grain sorting (phi)	1.31 ± 0.29		7386.3	1.74	0.003**
	Total carbon(%)	10.11 ± 1.90	Longitude,	5082.5	1.17	0.14
	Nitrogen (%)	0.08 ± 0.05	Mud content	8083.5	1.91	0.0007**
	Sulphur (%)	0.19 ± 0.06		5489.7	1.27	0.08
Geographic and bathymetry	Latitude (°S)	37.13 ± 0.62	Longitude, region	6691.1	1.56	0.01*
	Canyon defined by: central canyon axis			7260.6	0.82	0.96
	Canyon defined by: topographically distinct interior			4200.2	0.96	0.54
Gear performance	Grab weight (kg)	7.15 ± 4.51		5274.7	1.21	0.11

Correlated variables are those with a Pearson correlation >0.8 with the variable used in the analysis. Significant differences are indicated by

* (p<0.05) or

** (p<0.01).

**Fig 9:**
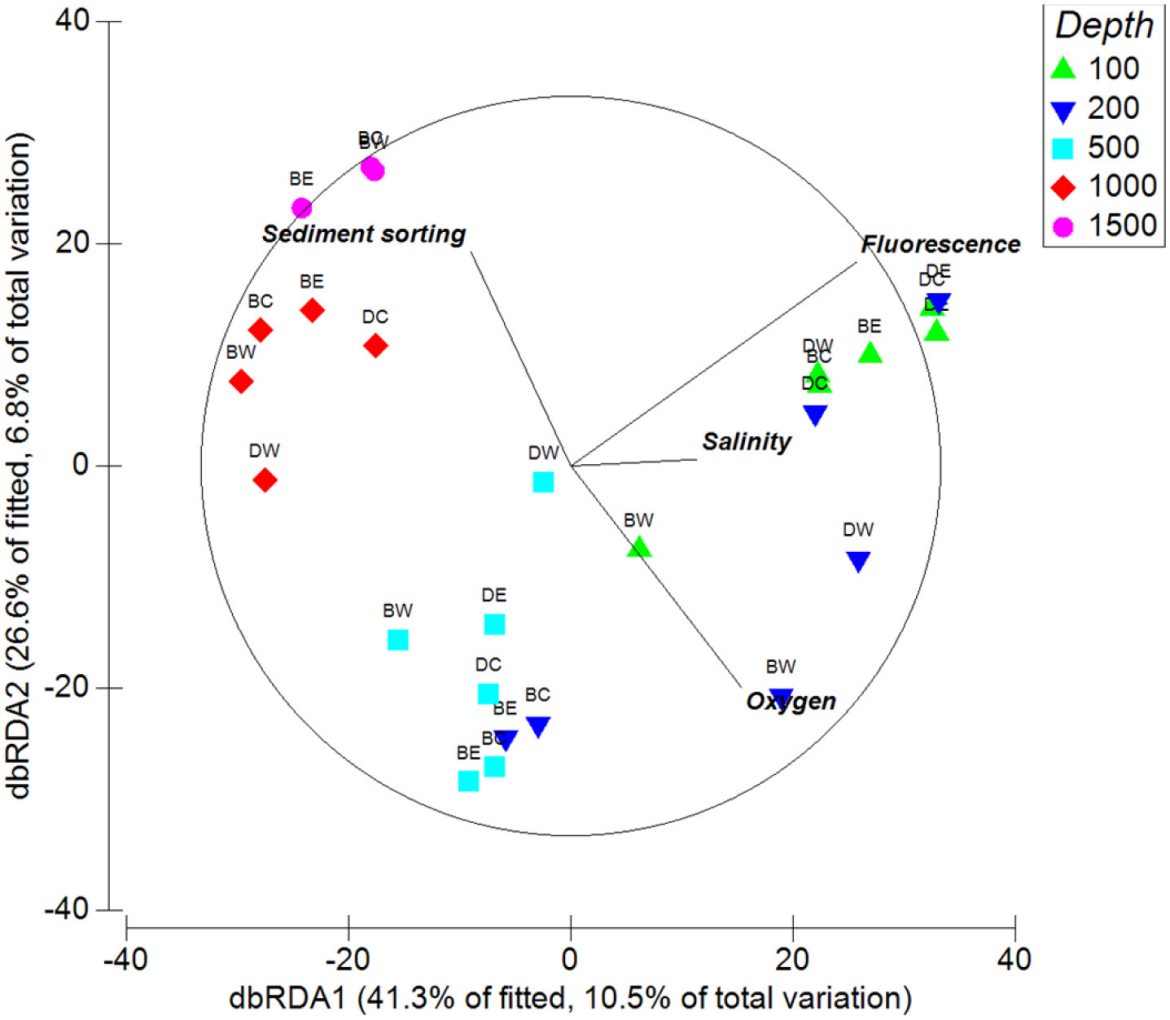
Constrained ordination of community composition by distance-based redundancy analysis (db RDA) with a vector overlay of predictor variables that significantly correlate with patterns of community composition in Table 7. Station codes are as in Fig 2.

While 10 of the 13 variables contributed to 49.3% of the variation in community composition, salinity, fluorescence, sediment sorting and oxygen content collectively contributed to 25.4%. Fitting further variables to a distance-based linear model (DISTLM) was not statistically significant (p>0.05), thus producing a parsimonious model with these four variables. The constrained ordination of this model by distance-based redundancy analysis (db RDA) graded community composition over 100–1000 m along the first axis and over 500–1500 m along the second axis (Fig 9). The first two axes accounted for 67.9% of the variation in the fitted model and 17.3% of the total variation.

## Discussion

The regional comparisons in this study are strongly influenced by shelf species since 70–74% of the species collected in the du Couedic and Bonney regions were shelf-restricted and only 7% occurred on both the shelf and slope. However, the sampling design was established with the purpose of comparing community structure inside each canyon with its same-depth counterpart and was only limited by the collecting capabilities of the gear, which failed at some of the 1000 and 1500 m stations in the du Couedic region due to the steep topography on the slope here. Where collections were successful, gear performance did not define biotic variation as much as bottom water and surface sediment variables. Best defining variables on the shelf were primary production (measured as fluorescence), nutrients, salinity and temperature. Sediment grain size and sorting and bottom water oxygen, temperature and nutrients defined biotic variation on the slope.

The high species number (531), given the relatively low sampling effort (27 samples, each 0.1m^2^) and the relatively coarse mesh size which excluded macrofauna <1.0 mm indicates that this area supports a high biodiversity. This corresponds with other observations of high species richness on the SE Australian coast (e.g., 803 species ≥0.5 mm at 11–51 m collected from 104 samples, each0.1m^2^ [62]). Compared to the adjacent eastern Great Australian Bight, where 240 macrofaunal species ≥1.0 mm were collected on the shelf to 200 m over 65 samples, each 0.1m^2^ [34], species richness in this study is nearly double (423 species on the shelf) with nearly a third the sampling effort [34]. In the du Couedic and Bonney regions, 71% and 66% of the species captured were found at only one station and in both regions, only 5% were captured in more than 3 stations. A large number of singletons was also reported by [34] and [62]. The Chao 1 index asymptotes slowly when there are many species with low incidences [63] as there were in this study. The increases in mean and variation of estimated species richness at the upper ends of the curves indicated that the estimates were reaching the limits of the data [63]. In addition to the low sampling effort, a contributing factor to the large number of rare species in this study was possibly also the relatively large distances between the stations (up to ≈20 km between same-depth stations) particularly on the shelf, which was necessitated by its shallow slope and the dimensions of the canyons.

High biomass samples resulted from the capture of sponges which were mostly confined to the shelf at 100–200 m [26]. High sponge biomass also occurs on the South and West Australian shelves [64–66]. By abundance and species richness, the annelids (mostly polychaetes) and arthropods (mostly peracarid crustaceans) were the consistent dominants at all depths, as in [34; 62]. The relationship of abundance to biomass of these solitary organisms (i.e., annelids, molluscs, arthropods, echinoderms and other solitary species) did not show a decline with depth. This indicates that the proportion of small organisms in the samples did not change with depth. Therefore, the relatively coarse mesh size in this study was not removing a disproportionately large fraction of small-bodied species at greater depth. The relatively higher percentage of Australian annelids found in this study compared to the other major taxa reflects the soft sediment and slope target of this study along with a macrofaunal focus, which would favour annelids. High taxonomic distinctness regardless of depth indicated a phyletically varied fauna even if species richness was low. The taxonomic distinctness measure is not affected by the small number of individuals at the deeper stations as it has been demonstrated to be unaffected by sample size [51; 52]. Evenness of abundance distribution among the species was also high both on the shelf and on the slope. Dominance increases were found at 500 m inside both canyons, however, and these were associated with increased sediment coarseness, suggestive of heightened currents.

### Hypothesis 1

By far the strongest pattern inside and outside of the canyons could be accounted for by depth and associated water masses (H1) with the annelids and arthropods primarily driving changes in composition. These strong gradients correspond with fish and megafaunal gradients noted by [26; 29]. Although upwelling, downwelling, eddies, density currents, winds and El Niño events alter the flows of the South Australian, Flinders and Antarctic Intermediate Water masses, particularly on the shelf [18] the strong faunal associations with these masses suggest a strong water mass effect. A similar depth gradient occurs in Western Australian polychaetes and crustaceans with highest species richness where seasonal upwelling occurs [67]. Water mass-associated changes in macroalgae were also observed by [68; 69].

The unconstrained MDS ordination showed a gradient of change in community composition regardless of transect location. Of all the same-depth samples, highest community resemblance was among the three du Couedic samples at 200 m. This was despite being 20 km apart. A similar closeness in community composition occurred between two of the three Bonney samples at 200 m although these were closer geographically (10 km). This may be due to a unifying effect on community composition of conditions close to the shelf break. Much less same-depth resemblance occurred at 100 m despite being 10 km apart and this may be due to the variable presence of sponge reefs. The lower resemblances among samples at 1000 and 1500 m are due to a sparseness of fauna. Species richness was nearly an order of magnitude less than on the shelf. Those that were found were mostly deep species not present shallower (e.g. loveniid heart urchins and species of lysianassid amphipods and among the polychaetes, the amphinomid *Linopherus* sp., the maldanid *Axiothella* sp., the opheliid *Antiobactrum* sp. and the spionid *Aurospio* sp.). The high evenness and taxonomic distinctness indicated that, even if few in number, species at all depths showed varied levels of relatedness and little dominance.

### Hypothesis 2

Community composition showed regional differences that were only marginally above significance at p<0.5 (H2). This was driven by the large proportion of unique species; 59% and 62% of those captured were unique to the du Couedic and Bonney regions, respectively. Fish distributions [28] and megafauna [26] both define the du Couedic and Bonney regions as being in different biotic provinces and the macrofaunal distributions in this study support this. The similarities and differences found here emphasize the need for updated bioregionalisation based on a larger sample of biotic groups and for habitat heterogeneity and geomorphic features to be included when surrogates for marine biodiversity are being developed [28; 70].

The much greater biomass variation on the shelf than deeper was primarily due to whether sponges were caught. These are the megafaunal biomass dominants on the South Australian shelf and reach high diversity and biomass where the bottom substrate is variable [26; 64; 71]. Sponges influence macrofaunal community composition by providing habitat for inquilines (e.g., among the inquilinous amphipods, a diversity of sebids, stenothoids, thaumatelsonids, colomastigids and leucothoids were found with the sponges). Solid attachment sites for sponges are also used by other sedentary organisms such as hydroids and bryozoans, which then provide habitat for nestlers (e.g. corophiid, ischyrocerid and caprellid amphipods in this study). The overall high abundance, biomass and diversity of the shelf macrofauna in both regions are attributable to inshore processes as well as from annual summer upwelling events that establish along Australian southern shelves [10; 18; 25; 26; 72]. Indeed, a large-scale surface upwelling event recorded at the time of sampling covered at least 50 km to either side of Bonney Canyon [29]. Upwelled water can move 200–400 km to the west within 10 days [18], thus providing nutrient-rich water to the entire shelf in this study.

### Hypothesis 3

From the perspective of canyon topography, the canyon interiors did not significantly differ from the exteriors (H3). However, the canyons can enable intrusion of deep fauna and bottom water up-slope and onto the shelf. Du Couedic Canyon penetrates 20 km into the shelf and essentially provides a deep water habitat incised into the shelf. This habitat is available for use by deep water species for feeding, reproduction or range expansion. Shelf-incising canyons also provide an opportunity for deep water exploitation without having to move off the shelf. Australia’s southern canyons are increasingly becoming a target for fishing [64; 73].

The sparse community within the canyons at 1000–1500 m depth is typical of V-shaped canyons, which typically have negative effects on macrofaunal benthos [5]. It is possible that different abundance contrasts would have been found if the two canyons had been sampled through their full length, which extends to the abyssal plain at 3000–5000 m. The lower part of du Couedic Canyon contains slump accumulations of shelf edge sediments [14]. Deep-sea faunal abundance can increase substantially in depositional sections of canyons (S3 Table).

Despite the marked drop in faunal abundance within the canyon incision, it is notable that species abundances at the canyon heads were high. There was a near tripling of abundance relative to comparative samples outside the canyon heads and a 1.5-2x increase in species richness. Only biomass was more variable and this was due to the variable presence of sponges. Many other canyons have shown similar contrasts in macrofaunal density relative to their exteriors, with significantly higher numbers found at the heads of Scripps/La Jolla and Mississippi Canyons [19; 74] (S3 Table) and also where descending particulates collect, such as in Kaikoura Canyon [5]. Such canyons must have their heads in coastal embayments with high loads of terrestrial material, be U-shaped in cross-section and have substantial inputs of coastal sediments [5]. An estimated 15% of the world’s submarine canyons may support biological hotspots world-wide [5]. Australian canyons do not receive any significant quantities of riverine sediment currently [17] and so any biological enrichment (apart from sediment enrichment during Pleistocene lowstands) must be explained by other mechanisms. The explanatory mechanism for fertilization of the du Couedic Canyon axis on the shelf may be organically rich density currents [26]. The inshore Spencer Gulf seasonally releases rich, high salinity water which flows through the upper du Couedic Canyon [18], and this is thought to be the mechanism for increased sponge biomass on the shelf in the du Couedic Canyon axis compared to the shelf inshore of the Bonney Canyon axis [26]. Such density currents are thought to flush submarine canyons widely on both high- and low-latitude continental margins [11]. Submarine canyons also collect planktonic food resources [4] and detritus from inshore macroalgae and seagrass populations [74], both of which can be dense along the South Australian coast [75; 76].

Both canyons showed greater species dominance in their upper reaches at 500 m than outside the canyons to either west or east. This can be explained by a greater proportion by abundance of sea cucumbers inside du Couedic Canyon and a different annelid composition dominated by oligochaetes and cirratulid, dorvilleid and spionid polychaetes. In Bonney Canyon, the oweniid polychaete *Myrioglobula* sp. drove the dominance within the canyon. The commonality is the high proportion of deposit feeders in the upper canyon reaches which was mostly caused by polychaetes. *Prionospio* sp., a polychaete that both surface deposit and suspension feeds, was one of the canyon head dominants. This genus was also a dominant in parts of the Portuguese canyons [31] (S3 Table). Species in this genus are typically opportunistic and able to colonize recently disturbed areas quickly [77]. Another opportunistic polychaete found in the Portunguese Nazaré Canyon was *Cossura* sp., thought to be aggregating on sedimented organic matter which was intercepted by the canyon from lateral shelf transport of terrestrial material [78]. Other opportunistic species found in Scripps/La Jolla Canyon were nebalian crustaceans and capitellid polychaetes, both of which are typical of rich organic matter ([79] and references therein). A species that was particularly abundant at the head of Mississippi Canyon was the tubicolous amphipod *Ampelisca mississippiana* [80]. This is also a mixed suspension-deposit feeder and different species of *Ampelisca* form dense mats where there is high pelagic-benthic coupling (for a review, see [81]). *Cossura* sp., nebalians, capitellids and *Ampelisca* sp. also occurred in the du Couedic and Bonney regions but not in elevated numbers at the canyon heads, indicating that different environmental conditions existed in these Australian canyons compared to the northern hemisphere canyons.

### Summary

We hypothesized that the macrofauna would (H1) vary with depth and water mass, (H2) differ between the two regions and (H3) differ between the canyon contours. With regard to H1, we found a significant difference in species composition between the shelf-confined South Australian, the upper slope Flinders and the lower slope Antarctic Intermediate Water masses. Abundance, biomass and species richness declined with depth although evenness and taxonomic distinctness remained high. The two regions differed in community composition (H2) but with a significance marginally above p = 0.5. Neither canyon differed significantly between their interior and exterior regardless of definition of the extent of the interior (H3). However, the canyon heads had higher macrofaunal abundance, species richness and/or biomass in their interiors compared to outside, suggesting an enrichment effect. Either at the canyon head (du Couedic) or the canyon’s upper reaches (Bonney), dominance was also higher than at equivalent depth outside the canyons and the substrate was coarser, indicating heightened current speed, presumably created by upwelling and downwelling within the canyons. Surface suspension and deposit feeding polychaetes such as oweniids and spionids were favoured in these upper reaches. A depauperate fauna within the lower reaches of the canyons is typical of V-shaped canyons [5] but it is possible that had these canyons been sampled through their full extent, enrichment effects would have been found in the deeper, depositional parts [31] (S3 Table). Adding the results of [29], who found strong water mass associations in the fish of Bonney Canyon, du Couedic Canyon could provide a channel for deep water fish to move onto the shelf while still within a deep water mass. Australian canyons are increasingly a target for trawling, which modifies pelagic and benthic food webs and increases canyon turbidity, resulting in impacts well beyond the trawled area range [73; 82]. Collectively, du Couedic and Bonney Canyons warrant further assessment for protection from resource exploitation. They support a highly diverse shelf ecosystem which is recognized as a potential Commonwealth Marine Reserve [83; 84] and which provides food resources and habitat for a number of critically endangered mammals including the Australian sea lion (*Neophoca cinerea)* and blue whale (*Balaenoptera musculus*) [85; 86].

## Supporting Information

S1 Data Canyons data: Primer-Permanova file; Taxon file; Environmental file; Factors; Biomass of major groups. (XLSX)

S1 Table Bottom water characteristics of the du Couedic and Bonney regions at the time of sampling. Pressure, temperature, salinity, fluorescence, PAR and oxygen were measured by CTD. Silicate, nitrate and phosphate levels were determined in the laboratory after water collection by Niskin bottle. Nd = not detectable.
(DOCX)

S2 Table Surface sediment characteristics of the du Couedic and Bonney regions at the time of sampling. (DOCX)

S3 Table Comparison of maximal macrofaunal densities inside and outside submarine canyons in this and other studies, ordered by depth. Species richness is also given at these high density locations. Dominant fauna, ordered by relative abundance, are coded A: amphipod crustacean; B: bivalve mollusc; C: chaetodermatid mollusc; Cu: cumacean crustacean; F: agglutinated foraminiferan; H: holothuroid echinoderm; I: isopod crustacean; N: nebalian crustacean; P: polychaete; S: scaphopod mollusc; T: tanaid crustacean. If more than one comparison was available, peak densities are given.
(DOCX)
