# Supplementary material for: Macrofaunal Patterns in and around du Couedic and Bonney Submarine Canyons, South Australia
Source: PLoS One. 2015 Nov 30;10(11):e0143921. doi: 10.1371/journal.pone.0143921 (PMC4664417; doi:10.1371/journal.pone.0143921)
Supplement: S1 Table — Pressure, temperature, salinity, fluorescence, PAR and oxygen were measured by CTD. Silicate, nitrate and phosphate levels were determined in the laboratory after water collection by Niskin bottle. Nd = not detectable. (DOCX) [file pone.0143921.s002.docx]

**S1 Table. Bottom water characteristics of the du Couedic and Bonney regions at the time of sampling.**

| Station | Latitude ( ̊S) | Longitude ( ̊E) | Day (Feb 2008) | Depth (m) | Temperature  ( ̊C) | Sa  lin  ity  (psu) | Fluores  cence  (μgl^-1^) | PAR  (volts) | Oxygen  (μMl^-1^) | Silicate  (μMl^-1^) | Nitrate (μMl^-1^) | Phosphate (μMl^-1^) |
| --- | --- | --- | --- | --- | --- | --- | --- | --- | --- | --- | --- | --- |
| du Couedic | | | | | | | | | | | | |
| \| DW 100 \| 36 14.460 \| 136 26.496 \| \| 18 \| \| 112 \| \| 10.29 \| \| 34.85 \| \| 14.37 \| \| \| 1.06 \| \| \| 244.98 \| \| \| \| \| 3.45 \| \| \| \| 14.35 \| \| 0.95 \| \| \| \| --- \| --- \| --- \| --- \| --- \| --- \| --- \| --- \| --- \| --- \| --- \| --- \| --- \| --- \| --- \| --- \| --- \| --- \| --- \| --- \| --- \| --- \| --- \| --- \| --- \| --- \| --- \| --- \| --- \| --- \| --- \| --- \| \| DC 100 \| 36 16.967 \| 136 32.161 \| \| 17 \| \| 113 \| \| 11.08 \| \| 34.98 \| \| 15.54 \| \| \| 0.71 \| \| \| 244.15 \| \| \| \| \| 2.73 \| \| \| \| 11.95 \| \| 0.81 \| \| \| \| DE 100 \| 36 20.137 \| 136 37.363 \| \| 17 \| \| 107 \| \| 11.60 \| \| 35.06 \| \| 14.82 \| \| \| 0.51 \| \| \| 248.03 \| \| \| \| \| 2.21 \| \| \| \| 10.24 \| \| 0.71 \| \| \| \| DW 200 \| 36 30.326 \| 136 18.444 \| \| 19 \| \| 154 \| \| 12.64 \| \| 35.22 \| \| 12.01 \| \| \| 0.19 \| \| \| 251.60 \| \| \| \| \| 1.06 \| \| \| \| 5.94 \| \| 0.57 \| \| \| \| DC 200 \| 36 23.495 \| 136 29.178 \| \| 18 \| \| 186 \| \| 11.21 \| \| 35.00 \| \| 13.16 \| \| \| nd \| \| \| 247.14 \| \| \| \| \| 2.22 \| \| \| \| 10.76 \| \| 0.97 \| \| \| \| DE 200 \| 36 33.173 \| 136 30.800 \| \| 19 \| \| 168 \| \| 11.22 \| \| 35.00 \| \| 15.49 \| \| \| 0.17 \| \| \| 246.58 \| \| \| \| \| 2.38 \| \| \| \| 10.91 \| \| 0.91 \| \| \| \| DW 500 \| 36 31.855 \| 136 17.313 \| \| 20 \| \| 442 \| \| 9.52 \| \| 34.72 \| \| 10.97 \| \| \| nd \| \| \| 249.95 \| \| \| \| \| 3.88 \| \| \| \| 15.94 \| \| 1.31 \| \| \| \| DC 500 \| 36 27.365 \| 136 27.450 \| \| 19 \| \| 494 \| \| 8.98 \| \| 34.64 \| \| 10.34 \| \| \| nd \| \| \| 249.07 \| \| \| \| \| 5.09 \| \| \| \| 17.58 \| \| 1.41 \| \| \| \| DE 500 \| 36 34.656 \| 136 29.820 \| \| 20 \| \| 486 \| \| 9.42 \| \| 34.71 \| \| 10.20 \| \| \| nd \| \| \| 250.72 \| \| \| \| \| 4.05 \| \| \| \| 16.18 \| \| 1.32 \| \| \| \| DW 1000 \| 36 32.614 \| 136 16.941 \| \| 21 \| \| 904 \| \| 5.22 \| \| 34.39 \| \| 9.80 \| \| \| nd \| \| \| 199.61 \| \| \| \| \| 30.30 \| \| \| \| 29.39 \| \| 2.23 \| \| \| \| DC 1000 \| 36 32.126 \| 136 24.555 \| \| 20 \| \| 824 \| \| 5.09 \| \| 34.39 \| \| 11.40 \| \| \| nd \| \| \| 197.79 \| \| \| \| \| 32.48 \| \| \| \| 29.74 \| \| 2.20 \| \| \| \| DE 1000 \| 36 35.546 \| 136 29.213 \| \| 20 \| \| 1009 \| \| 4.19 \| \| 34.40 \| \| 10.22 \| \| \| nd \| \| \| 192.28 \| \| \| \| \| 43.57 \| \| \| \| 31.61 \| \| 2.38 \| \| \| \| DW 1500 \| 36 33.563 \| 136 16.378 \| \| 21 \| \| 1626 \| \| 2.77 \| \| 34.56 \| \| 10.02 \| \| \| nd \| \| \| 170.97 \| \| \| \| \| 75.17 \| \| \| \| 33.85 \| \| 2.66 \| \| \| \| DC 1500 \| 36 34.834 \| 136 24.703 \| \| 21 \| \| 1475 \| \| 2.87 \| \| 34.54 \| \| 11.33 \| \| \| nd \| \| \| 172.24 \| \| \| \| \| 72.57 \| \| \| \| 33.98 \| \| 2.65 \| \| \| \| DE 1500 \| 36 36.216 \| 136 28.820 \| \| 21 \| \| 1450 \| \| 2.99 \| \| 34.51 \| \| 10.40 \| \| \| nd \| \| \| 173.96 \| \| \| \| \| 68.99 \| \| \| \| 33.19 \| \| 2.62 \| \| \| \| Bonney \| \| \| \| \| \| \| \| \| \| \| \| \| \| \| \| \| \| \| \| \| \| \| \| \| \| \| \| \| \| \| \| \| BW 100 \| 37 28.009 \| 139 30.927 \| \| 07 \| \| 102 \| \| 10.35 \| \| 34.86 \| \| 11.22 \| \| \| nd \| \| \| 253.88 \| \| \| \| \| 3.17 \| \| \| \| 12.94 \| \| 0.92 \| \| \| \| BC 100 \| 37 30.156 \| 139 36.900 \| \| 07 \| \| 102 \| \| 10.69 \| \| 34.91 \| \| 13.87 \| \| \| nd \| \| \| 252.69 \| \| \| \| \| 2.54 \| \| \| \| 10.78 \| \| 0.80 \| \| \| \| BE 100 \| 37 35.313 \| 139 38.916 \| \| 07 \| \| 99 \| \| 11.09 \| \| 34.98 \| \| 14.28 \| \| \| nd \| \| \| 251.53 \| \| \| \| \| 2.64 \| \| \| \| 11.04 \| \| 0.83 \| \| \| \| BW 200 \| 37 32.650 \| 139 27.605 \| \| 08 \| \| 160 \| \| 12.06 \| \| 35.14 \| \| 11.13 \| \| \| 0.06 \| \| \| 254.73 \| \| \| \| \| 1.46 \| \| \| \| 7.12 \| \| 0.60 \| \| \| \| BC 200 \| 37 36.054 \| 139 34.672 \| \| 08 \| \| 196 \| \| 9.65 \| \| 34.74 \| \| 10.33 \| \| \| 0.38 \| \| \| 249.91 \| \| \| \| \| 3.92 \| \| \| \| 15.88 \| \| 1.09 \| \| \| \| BE 200 \| 37 38.635 \| 139 37.286 \| \| 08 \| \| 182 \| \| 9.52 \| \| 34.73 \| \| 9.97 \| \| \| 0.10 \| \| \| 253.59 \| \| \| \| \| 4.05 \| \| \| \| 15.31 \| \| 1.08 \| \| \| \| BW 500 \| 37 34.553 \| 139 26.384 \| \| 09 \| \| 516 \| \| 8.29 \| \| 34.55 \| \| 9.92 \| \| \| nd \| \| \| 241.04 \| \| \| \| \| 6.57 \| \| \| \| 20.17 \| \| 1.34 \| \| \| \| BC 500 \| 37 38.519 \| 139 32.309 \| \| 09 \| \| 454 \| \| 8.92 \| \| 34.63 \| \| 10.34 \| \| \| nd \| \| \| 249.67 \| \| \| \| \| 4.68 \| \| \| \| 18.19 \| \| 1.19 \| \| \| \| BE 500 \| 37 42.432 \| 139 34.458 \| \| 08 \| \| 456 \| \| 9.02 \| \| 34.64 \| \| 9.93 \| \| \| nd \| \| \| 251.98 \| \| \| \| \| 4.16 \| \| \| \| 17.42 \| \| 1.16 \| \| \| \| BW 1000 \| 37 41.972 \| 139 20.773 \| \| 10 \| \| 1001 \| \| 4.06 \| \| 34.40 \| \| 9.72 \| \| \| nd \| \| \| 190.45 \| \| \| \| \| 46.98 \| \| \| \| 32.58 \| \| 2.30 \| \| \| \| BC 1000 \| 37 41.589 \| 139 28.994 \| \| 10 \| \| 951 \| \| 4.37 \| \| 34.39 \| \| 10.05 \| \| \| nd \| \| \| 194.15 \| \| \| \| \| 41.38 \| \| \| \| 32.03 \| \| 2.22 \| \| \| \| BE 1000 \| 37 47.700 \| \| 139 30.779 \| \| 09 \| \| 992 \| \| 4.17 \| \| 34.39 \| \| 10.70 \| \| \| nd \| \| \| 192.73 \| \| \| \| \| 45.29 \| \| \| \| 33.68 \| 2.28 \| \| \| \| BW 1500 \| 37 48.990 \| \| 139 15.641 \| \| 10 \| \| 1503 \| \| 2.75 \| \| 34.57 \| \| \| 10.95 \| \| \| nd \| \| \| 171.07 \| \| 75.54 \| \| \| \| 34.04 \| \| \| \| 2.48 \| \| BC 1500 \| 37 43.956 \| \| 139 26.253 \| \| 10 \| \| 1597 \| \| 2.66 \| \| 34.60 \| \| \| 10.73 \| \| \| nd \| \| \| 170.68 \| \| 82.07 \| \| \| \| 35.75 \| \| \| \| 2.50 \| \| BE 1500 \| 37 53.770 \| \| 139 26.572 \| \| 11 \| \| 1504 \| \| 2.72 \| \| 34.57 \| \| 9.97 \| \| \| nd \| \| \| 171.04 \| \| 78.00 \| \| \| \| 35.91 \| \| \| \| \| 2.50 \| | | | | | | | | | | | | |

Pressure, temperature, salinity, fluorescence, PAR and oxygen were measured by CTD. Silicate, nitrate and phosphate levels were determined in the laboratory after water collection by Niskin bottle. Nd = not detectable.
