# Supplementary material for: Macrofaunal Patterns in and around du Couedic and Bonney Submarine Canyons, South Australia
Source: PLoS One. 2015 Nov 30;10(11):e0143921. doi: 10.1371/journal.pone.0143921 (PMC4664417; doi:10.1371/journal.pone.0143921)
Supplement: S2 Table — (DOCX) [file pone.0143921.s003.docx]

**S2Table. Surface sediment characteristics of the du Couedic and Bonney regions at the time of sampling.**

| Station | Latitude ( ̊S) | Longitude ( ̊E) | Day (Feb 2008) | Grab Weight (kg) | Depth (m) | % <63μ | Mean (phi) | Sorting (phi) | Nitrogen  (%) | Sulphur (%) |
| --- | --- | --- | --- | --- | --- | --- | --- | --- | --- | --- |
| du Couedic | | | | | | | | | | |
| DW 100 | 36 14.487 | 136 26.431 | 18 | 10 | 120 | 2.48 | 0.74 | 1.13 | 0.06 | 0.26 |
| DC 100 | 36 17.024 | 136 32.248 | 18 | 4 | 114 | 2.36 | 1.14 | 1.10 | 0.06 | 0.22 |
| DE 100 | 36 20.166 | 136 37.345 | 18 | 5 | 106 | 3.33 | 1.73 | 1.21 | 0.06 | 0.25 |
| DW 200 | 36 30.326 | 136 18.361 | 19 | 2.5 | 154 | 4.18 | 2.67 | 1.07 | 0.05 | 0.20 |
| DC 200 | 36 23.500 | 136 29.196 | 18 | 8 | 186 | 2.79 | 1.69 | 1.30 | 0.06 | 0.21 |
| DE 200 | 36 33.220 | 136 30.305 | 19 | 4.5 | 169 | 8.57 | 2.80 | 1.18 | 0.06 | 0.22 |
| DW 500 | 36 31.790 | 136 17.452 | 20 | 9 | 388 | 31.84 | 3.64 | 1.85 | 0.08 | 0.28 |
| DC 500 | 36 27.430 | 136 27.493 | 19 | 13 | 500 | 2.65 | 0.79 | 1.06 | 0.01 | 0.20 |
| DE 500 | 36 34.614 | 136 29.890 | 20 | 12 | 482 | 49.93 | 4.21 | 1.42 | 0.19 | 0.25 |
| DW 1000 | 36 32.684 | 136 16.989 | 21 | 1.5 | 954 | 43.09 | 4.13 | 1.28 | 0.14 | 0.20 |
| DC 1000 | 36 32.230 | 136 24.572 | 20 | 0.5 | 851 | 26.40 | 3.78 | 1.27 | 0.13 | 0.27 |
| DE 1000 |  |  |  | - | - | - | - | - | - | - |
| DW 1500 |  |  |  | - | - | - | - | - | - | - |
| DC 1500 | 36 34.842 | 136 25.042 | 21 | 0.5 | 1483 | 43.66 | 4.08 | 1.44 | 0.10 | 0.35 |
| DE 1500 |  |  |  | - | - | - | - | - | - | - |
| Bonney | | | | | | | | | | |
| BW 100 | 37 28.026 | 139 31.291 | 07 | 2 | 104 | 1.23 | 1.44 | 1.43 | 0.04 | 0.20 |
| BC 100 | 37 30.151 | 139 36.944 | 07 | 10 | 103 | 0.97 | 0.09 | 1.33 | 0.06 | 0.13 |
| BE 100 | 37 35.546 | 139 39.438 | 07 | 3.5 | 100 | 2.34 | 0.15 | 1.32 | 0.04 | 0.17 |
| BW 200 | 37 32.665 | 139 27.419 | 08 | 7.5 | 160 | 1.90 | 2.37 | 0.87 | 0.01 | 0.13 |
| BC 200 | 37 36.026 | 139 34.410 | 08 | 9.5 | 200 | 2.04 | 2.35 | 0.96 | 0.01 | 0.15 |
| BE 200 | 37 38.630 | 139 37.156 | 08 | 11 | 192 | 1.82 | 1.66 | 1.07 | 0.01 | 0.15 |
| BW 500 | 37 34.570 | 139 26.365 | 09 | 10 | 520 | 13.52 | 2.73 | 1.30 | 0.09 | 0.18 |
| BC 500 | 37 38.523 | 139 32.311 | 09 | 5 | 450 | 5.93 | 2.46 | 0.83 | 0.06 | 0.16 |
| BE 500 | 37 42.438 | 139 34.486 | 08 | 8 | 456 | 12.46 | 3.05 | 0.92 | 0.09 | 0.16 |
| BW 1000 | 37.41.991 | 139 20.853 | 10 | 11 | 1003 | 56.07 | 4.23 | 1.57 | 0.13 | 0.26 |
| BC 1000 | 37 41.615 | 139 28.983 | 10 | 17 | 952 | 54.43 | 4.01 | 1.81 | 0.16 | 0.14 |
| BE 1000 | 37 47.698 | 139 30.771 | 09 | 13 | 992 | 52.57 | 4.14 | 1.57 | 0.13 | 0.15 |
| BW 1500 | 37 49.021 | 139 15.643 | 10 | 11 | 1508 | 56.34 | 4.23 | 1.58 | 0.11 | 0.09 |
| BC 1500 | 37 43.957 | 139 26.209 | 11 | 2.5 | 1593 | 56.61 | 4.23 | 1.68 | 0.16 | 0.16 |
| BE 1500 | 37 53.818 | 139 26.544 | 11 | 1.5 | 1505 | 48.45 | 3.84 | 1.82 | 0.12 | 0.11 |
