# Supplementary material for: Macrofaunal Patterns in and around du Couedic and Bonney Submarine Canyons, South Australia
Source: PLoS One. 2015 Nov 30;10(11):e0143921. doi: 10.1371/journal.pone.0143921 (PMC4664417; doi:10.1371/journal.pone.0143921)
Supplement: S3 Table — Species richness is also given at these high density locations. Dominant fauna, ordered by relative abundance, are coded A: amphipod crustacean; B: bivalve mollusc; C: chaetodermatid mollusc; Cu: cumacean crustacean; F: agglutinated foraminiferan; H: holothuroid echinoderm; I: isopod crustacean; N: nebalian crustacean; P: polychaete; S: scaphopod mollusc; T: tanaid crustacean. If more than one comparison was available, peak densities are given. (DOCX) [file pone.0143921.s004.docx]

**S3 Table. Comparison of maximal macrofaunal densities inside and outside submarine canyons in this and other studies, ordered by depth.**

| Canyon | Region | Location within the canyon | Depth (m) | Maximum abundance inside (mean ± s.e. ind m^-2^) | Same-depth abundance outside (mean ± s.e. ind m^-2^) | Species richness inside (mean ± s.e. sampling unit^-1^) | Species richness outside (mean ± s.e. sampling unit^-1^) | Separation dist-ance (km) | Macrofaunal size range (mm) | Dominant fauna (at this location) | Sampler size (m^2^) | Sampler type | Reference | Comment |
| --- | --- | --- | --- | --- | --- | --- | --- | --- | --- | --- | --- | --- | --- | --- |
| Newport | NE Pacific: California | Head | 60 | 3490 ± 1996 (sd) (n=20) | 4575 ± 1681 (sd) (n=20) | 48.7 ± 11.5  (n = 20) | 107 ± 17  (n = 20) | 4 | ≥1 |  | 0.1 | Van Veen grab | [86] |  |
| La Jolla | NE Pacific: California | Head | 15-300 | 3,240,000 |  |  |  |  |  | *Nebalia* spp. (N), *Orchomene limodes* (A), *Aoroides spinosus* (A) |  |  | [74] | High numbers inside were attributed to sampling detrital patches of surfgrass and kelp |
| Scripps/La Jolla | NE Pacific: California | Head | 100 | 59,900 (n=8) | 3200 (n=4) | 53 ± 14 | 64 ± 26 | ≈5 | ≥0.3 | *Nebalia* sp. (N), *Orchomene limodes* (A), *Capitella* cf *capitata* (P) | 0.1 | Slurp-gun | [19] | Interpreted from Fig. 5 (abundance) and 11 (species richness) |
| du Couedic | Indian Ocean**:** SE Australia | Head | 186 | 1328 | 592.5 ± 74.5 (n=2) | 77 | 47.5 ± 10.6 (n=2) | 19.5 | ≥1 | *Prionospio* sp. (P), *Lumbrineris* sp. (P), *Myrioglobula* sp. (P), *Leptoecia* sp. (P), *Melinoides* sp. (P) | 0.1 | Smith Macintyre grab | This study | Separation distance average of the distance of DC 200 to DW 200 and DE 200. Dominants have abundance at DC 200 ≥40 ind m^-2^ |
| Bonney | Indian Ocean: SE Australia | Head | 450 | 1190 | 435 ± 15 (n=2) | 38 | 25.5 ± 3.5 (n=2) | 9.5 | ≥1 | *Myrioglobula* sp. (P), *Aphelochaeta* sp. (P), Lysiannasid sp. (A), *Hemipodia* sp. (P), *Levinsenia* sp. (P), Spionidae sp. (P) | 0.1 | Smith Macintyre grab | This study | Separation distance average of the distance of BC 500 to BW 500 and BE 500. Dominants have abundance at BC 500 ≥40 ind m^-2^ |
| Mississippi | Gulf of Mexico | Head | 480 | 15,571 (n=5) |  |  |  |  | ≥0.3 | *Ampelisca mississippiana* (A) | 0.2 | Box corer | [87] |  |
| Soquel/  Monterey | NE Pacific: California | 6 and 50 m from cliff base | 595 | 584 ± 0 (n=2) |  | 14 ± 1.4 (n=2) |  |  | ≥0.25 | Polychaetes | 0.385 | Corer | [30] | Maximum abundance interpreted from Fig. 4 |
| Nazaré | NE Atlantic: Portugal | Upper | 897 | 474.5 ± 102.0 (n=2) | 191.3 ± 48.5 | 37 | 35 | 87 | ≥0.5 | *Carangoliopsis spinulosa* (A), *Paradiopatra hispanica* (P), cf. *Colletea* sp. (T), *Abyssoninoe abyssorum* (P), Scaphopoda (S), Chaetodermomorpha (C) | 0.196 | NIOZ circular box corer | [31] | Only location where comparative samples outside were taken. Peak abundance was in the Middle location |
| Setúbal | NE Atlantic: Portugal | Upper | 970 | 385.2 ± 63.8 (n=2) | 188.8 ± 29.5 | 50 | 50 | 60 | ≥0.5 | *Phylamphicteis* sp. (P), *Prionospio sandersi* (P), *Carangoliopsis spinulosa* (A), Maldanidae spp. (P), Apseudidae (T) | 0.196 | NIOZ circular box corer | [31] |  |
| Cascais | NE Atlantic: Portugal | Upper | 978 | 583.3 ± 19.2 (n=3) | 188.8 ± 29.5 | 70 | 50 | 70 | ≥0.5 | *Phylamphicteis* sp. (P), Polynoidae sp. (P), *Prionospio sandersi* (P), *Levinsenia* sp. (P), Maldanidae sp. (P), *Leucon* sp. (Cu) | 0.196 | NIOZ circular box corer | [31] | Only location where comparative samples outside were taken. Peak abundance was in the Middle location |
| Kaikoura | SW Pacific: New Zealand | Head | ≈1000 | 51,500 ± 5500 (n=10) | ≈25,000 |  |  | ≈167 | ≥0.3 |  | 0.08 | Multi-corer | [5] |  |
| Campeche | SW Gulf of Mexico | Middle | 2560 | 6925 ± 2258 (n=3) |  |  |  |  | ≥0.25 |  | 0.16 | USNEL Boxcorer | [88] |  |
| Whittard | NE Atlantic: UK | Upper part of west branch | 2735 | 2717 |  |  |  |  | ≥0.5 | *Saccorhiza* (F) | 0.2 | Box corer | [89] |  |
| Nazaré | NE Atlantic: Portugal | Middle | 2894 | 74,184 (n=1) |  | 42 |  |  | ≥0.5 | *Cossura* sp. (P) | 0.125 | 0.25 m^2^ USNEL box corer | [78] | Extrapolated from 9273 ind 0.125 m^-2^. High numbers were attributed to canyon interception of laterally transported terrestrial organic matter |
| Cascais | NE Atlantic: Portugal | Middle | 3199 | 1125.1± 138.0 (n=5) |  | 102 |  |  | ≥0.5 | *Thyasira* cf. *flexuosa* (B), *Siboglinum* cf. *angustum* (P), *Prionospio* sp. (P), *Macrostylis* cf. *abyssicola* (I) | 0.008+ | UKORS mega-corer | [31] | 5-8 0.008 m^2^ cores pooled to form one replicate sample |
| Setúbal | NE Atlantic: Portugal | Middle | 3224 | 2241.3 ± 164.2 (n=5) |  | 102 |  |  | ≥0.5 | *Prionospio* sp. (P), *Melinampharete* sp. (P), *Levinsenia gracilis (P)*, *Thyasira* sp. (B), *Haploniscus* cf. *charcoti* (I) | 0.008+ | UKORS mega-corer | [31] | 5-8 0.008 m^2^ cores pooled to form one replicate sample |
| Whittard | NE Atlantic: UK | East branch | 3427 | 6249 ± 1363 (sd) (n=5) | 2744 ± 269 (s d) (n=5) |  |  | 58 | ≥0.3 | Amphinomidae (P), Spionidae (P) | 0.008+ | Mega-corer | [22] | 5 0.008 m^2^ cores pooled to form one replicate sample |
| Nazaré | NE Atlantic: Portugal | Middle | 3517 | 4599.5 ± 441.6 (n=4) |  | 46 |  |  | ≥0.5 | *Prionospio* sp. (P), Scaphopoda (S), *Yoldiella* sp. (B), Pseudotanaidae sp. (T), *Ypsilothuria bitentaculata* (H) | 0.008+ | UKORS mega-corer | [31] | 5-8 0.008 m^2^ cores pooled to form one replicate sample |

Dominant fauna, ordered by relative abundance, are coded A: amphipod crustacean; B: bivalve mollusc; C: chaetodermatid mollusc; Cu: cumacean crustacean; F: agglutinated foraminiferan; H: holothuroid echinoderm; I: isopod crustacean; N: nebalian crustacean; P: polychaete; S: scaphopod mollusc; T: tanaid crustacean. If more than one comparison was available, peak densities are given.

**Additional References**

87. Maurer D, Robertson G, Gerlinger T. Comparison of community structure of soft-bottom macrobenthos of the Newport submarine canyon, California and the adjoining shelf. Int Rev Ges Hydrobio. 1994;79: 591-603.

88. Escobar-Briones E, Santillan ELE, Legendre P. Macrofaunal density and biomass in the Campeche Canyon, Southwestern Gulf of Mexico. Deep Sea Res Part 2 Top Stud Oceanogr. 2008;55: 2679-2685.

89. Duineveld G, Lavaleye M, Berghuis E, de Wilde P. Activity and composition of the benthic fauna in the Whittard Canyon and the adjacent continental slope (NE Atlantic). Oceanologica Acta. 2001;24: 69-83.
